# Supplementary material for: Rewiring of Aminoacyl-tRNA Synthetase Localization and Interactions in Plants With Extensive Mitochondrial tRNA Gene Loss
Source: Mol Biol Evol. 2023 Jul 18;40(7):msad163. doi: 10.1093/molbev/msad163 (PMC10375062; doi:10.1093/molbev/msad163)
Supplement: msad163_Supplementary_Data [file msad163_supplementary_data.zip › Supp.table1.pdf]

aaRS	Mapped TAIR gene	Localization in *A. thaliana*	Iso-Seq transcript coverage				
			Agrostemma	*S. conica*	*S. latifolia*	*S. noctiflora*	*S. vulgaris*
**AlaRS**	At1g50200	Cytosol/Mitochondria*/Plastid*	1084	786	2630	60	1617
**AlaRS**	At5g22800	Mitochondria*/Plastid*	189	228	143	9	365
**ArgRS**	At1g66530:At4g26300	Cytosol:Plastid*	190	139	265	54	511
**AsnRS**	At1g70980:At5g56680	Cytosol:Cytosol	135	85	206	42	208
**AsnRS**	At4g17300	Mitochondria*/Plastid*	68	48	67	14	40
**AsnRS**	At3g07420	Cytosol	23	0	0	4	2
**AspRS**	At4g26870:At4g31180	Cytosol:Cytosol	165	62	114	82	140
**AspRS**	At4g33760	Mitochondria*/Plastid*	44	37	37	6	61
**CysRS**	At3g56300:At5g38830:At2g31170	Cytosol:Cytosol:Mitochondria*/Plastid*	135	102	229	93	116
**GlnRS**	At1g25350	Cytosol	640	194	301	65	554
**GluRS**	At5g26710	Cytosol	139	510	342	97	268
**GluRS**	At5g64050	Mitochondria*/Plastid*	92	48	130	35	86
**GlyRS**	At1g29870:At1g29880:At3g44740	Cytosol:Mitochondria*:Cytosol	896	445	1225	90	552
**GlyRS**	At3g48110	Mitochondria*/Plastid*	114	73	165	6	225
**HisRS**	At3g02760	Cytosol	525	221	357	11	468
**HisRS**	At3g46100	Mitochondria*/Plastid*	62	23	36	19	29
**IleRS**	At4g10320	Cytosol	713	687	812	31	684
**IleRS**	At5g49030	?/Plastid*	472	258	498	6	331
**LeuRS**	At1g09620	Cytosol/Mitochondria*	1441	732	829	27	1672
**LeuRS**	At4g04350	Plastid*	90	27	77	2	94
**LysRS**	At3g13490	Mitochondria*/Plastid*	107	148	199	21	376
**LysRS**	At3g11710	Cytosol	172	102	501	61	314
**MetRS**	At3g55400	Mitochondria*/Plastid*	197	11	77	10	51
**MetRS**	At2g40660	Cytosol	53	13	19	33	26
**MetRS**	At4g13780	Cytosol	202	258	162	62	363
**PheRS**	At3g58140	Mitochondria*/Plastid*	56	22	33	23	35
**PheRS**	At1g72550	Cytosol	191	63	140	37	293
**PheRS**	At4g39280	Cytosol	81	118	161	69	165
**ProRS**	At3g62120	Cytosol	133	233	384	116	356
**ProRS**	At5g52520	Mitochondria*/Plastid*	48	51	73	45	64
**SerRS**	At5g27470	Cytosol	167	67	68	98	187
**SerRS**	At1g11870	Mitochondria*/Plastid*	35	3	20	0	17
**ThrRS**	At2g04842	Mitochondria*/Plastid*	120	155	65	53	135
**ThrRS**	At1g17960:At5g26830	Cytosol:Mitochondria*	390	238	384	66	184
**TrpRS**	At3g04600	Cytosol	29	21	24	32	47
**TrpRS**	At2g25840	Mitochondria*/Plastid*	19	8	5	6	18
**TyrRS**	At1g28350:At2g33840	Cytosol:Cytosol	326	12	97	14	189
