# Supplementary material for: Rewiring of Aminoacyl-tRNA Synthetase Localization and Interactions in Plants With Extensive Mitochondrial tRNA Gene Loss
Source: Mol Biol Evol. 2023 Jul 18;40(7):msad163. doi: 10.1093/molbev/msad163 (PMC10375062; doi:10.1093/molbev/msad163)
Supplement: msad163_Supplementary_Data [file msad163_supplementary_data.zip › Supp.table3.pdf]

Supplementary table 3| Yields from Iso-Seq libraries.

**Species**	**CCSs**	**FLNCs**	**Clusters (HQ)**
A. githago	2034058	2029458	153293
S. conica	1535114	1525890	94736
S. latifolia	1584689	1580565	122274
S. vulgaris	1765441	1762081	126377
