# Supplementary material for: Rewiring of Aminoacyl-tRNA Synthetase Localization and Interactions in Plants With Extensive Mitochondrial tRNA Gene Loss
Source: Mol Biol Evol. 2023 Jul 18;40(7):msad163. doi: 10.1093/molbev/msad163 (PMC10375062; doi:10.1093/molbev/msad163)

Cytosolic GlnRS

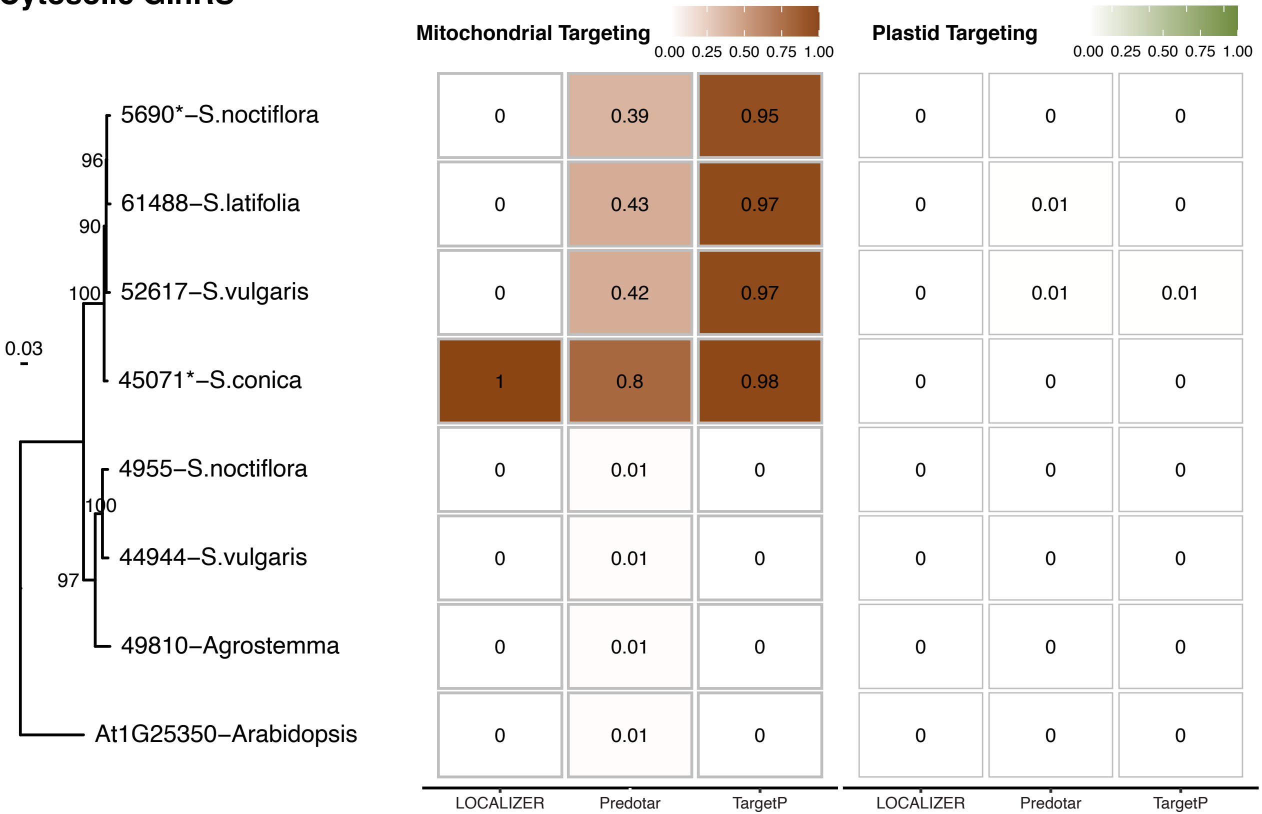

Supplement: msad163_Supplementary_Data [file msad163_supplementary_data.zip › Supp.fig6_GlnRS.pdf]
