# Supplementary figures and images for: Rewiring of Aminoacyl-tRNA Synthetase Localization and Interactions in Plants With Extensive Mitochondrial tRNA Gene Loss
Source: Mol Biol Evol. 2023 Jul 18;40(7):msad163. doi: 10.1093/molbev/msad163 (PMC10375062; doi:10.1093/molbev/msad163)

# Colors for amino acids

|                                                                                     |   |                                                                                     |     |
|-------------------------------------------------------------------------------------|---|-------------------------------------------------------------------------------------|-----|
| 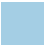   | m | 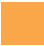   | w   |
| 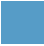   | s | 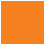   | t   |
| 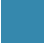   | k | 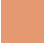   | g   |
| 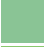   | l | 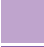   | e   |
| 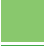   | i | 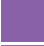   | r   |
| 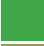  | h | 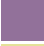  | a   |
| 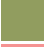 | f | 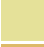 | v   |
| 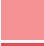 | y | 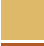 | c   |
| 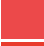 | n | 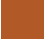 | d   |
| 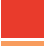 | q |                                                                                     |     |
| 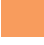 | p | 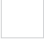 | gap |

Supplement: msad163_Supplementary_Data [file msad163_supplementary_data.zip › Supp.fig21_AA_key.pdf]

Cytosolic/Organellar AlaRS

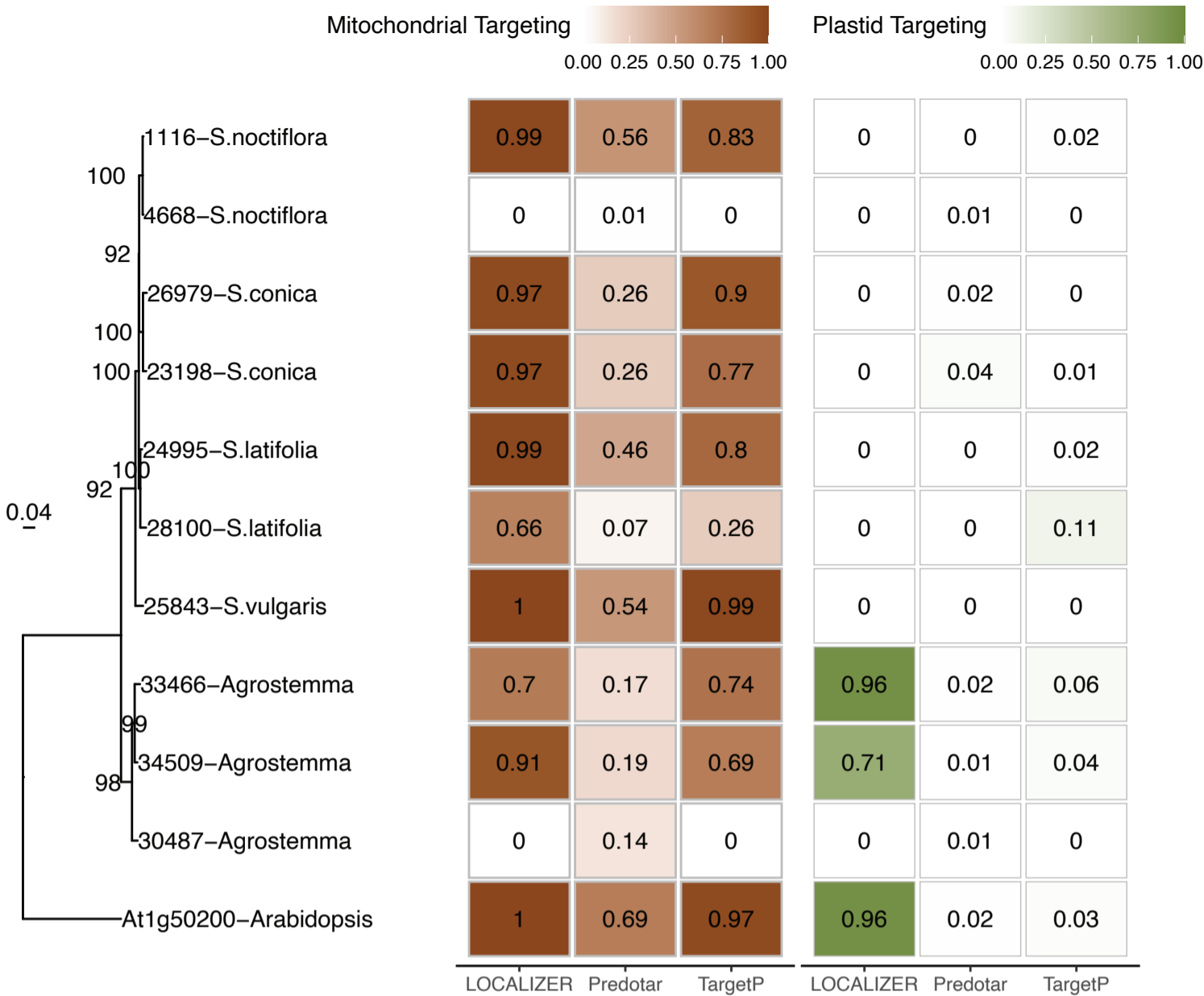

Supplement: msad163_Supplementary_Data [file msad163_supplementary_data.zip › Supp.fig1_AlaRS.pdf]

# Cytosolic/Organellar ArgRS

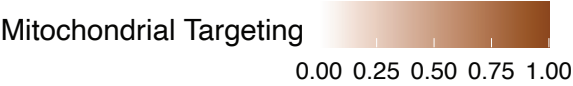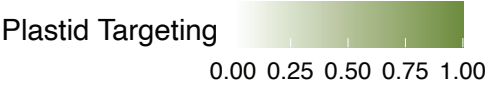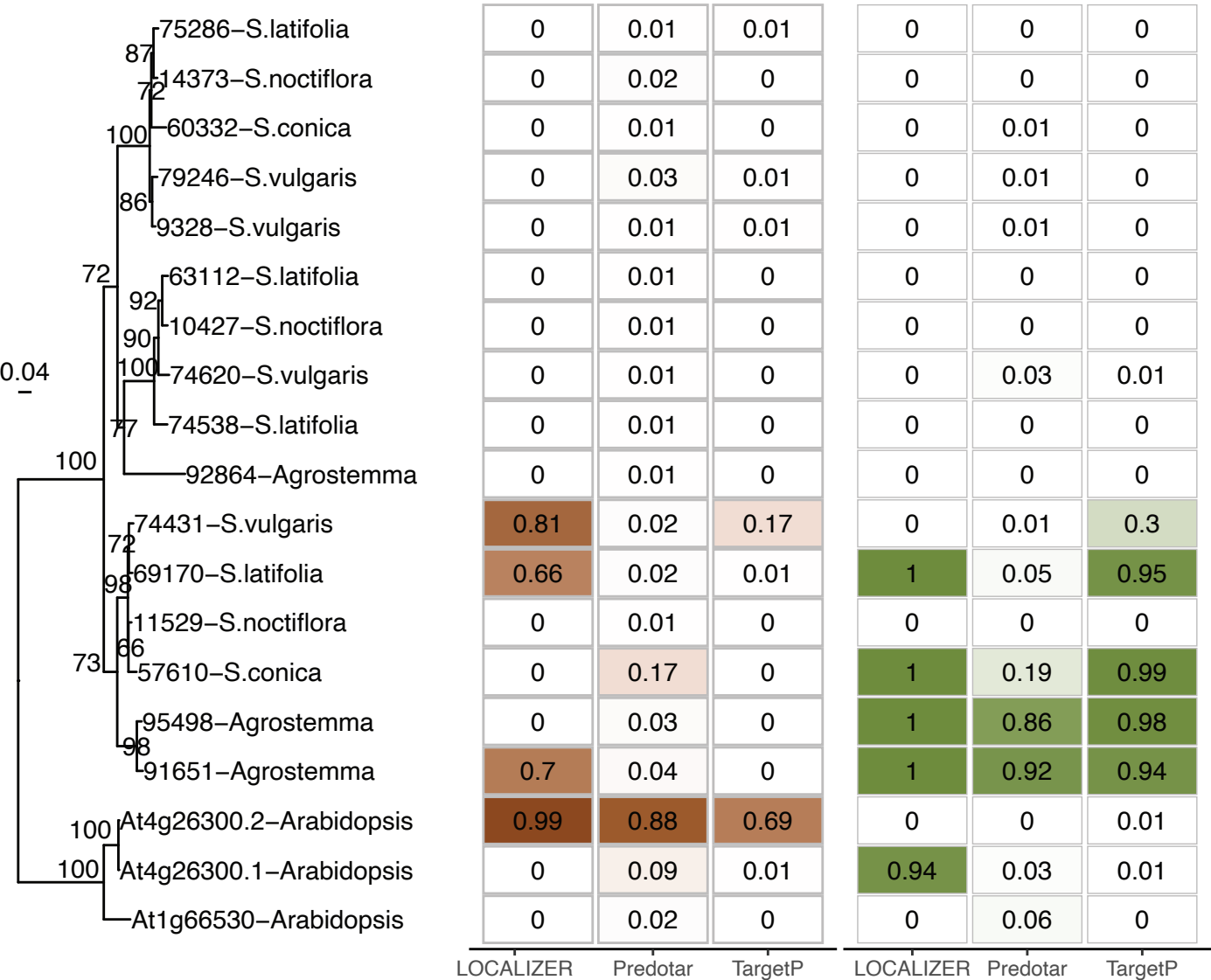

Supplement: msad163_Supplementary_Data [file msad163_supplementary_data.zip › Supp.fig2_ArgRS.pdf]

Cytosolic AsnRS

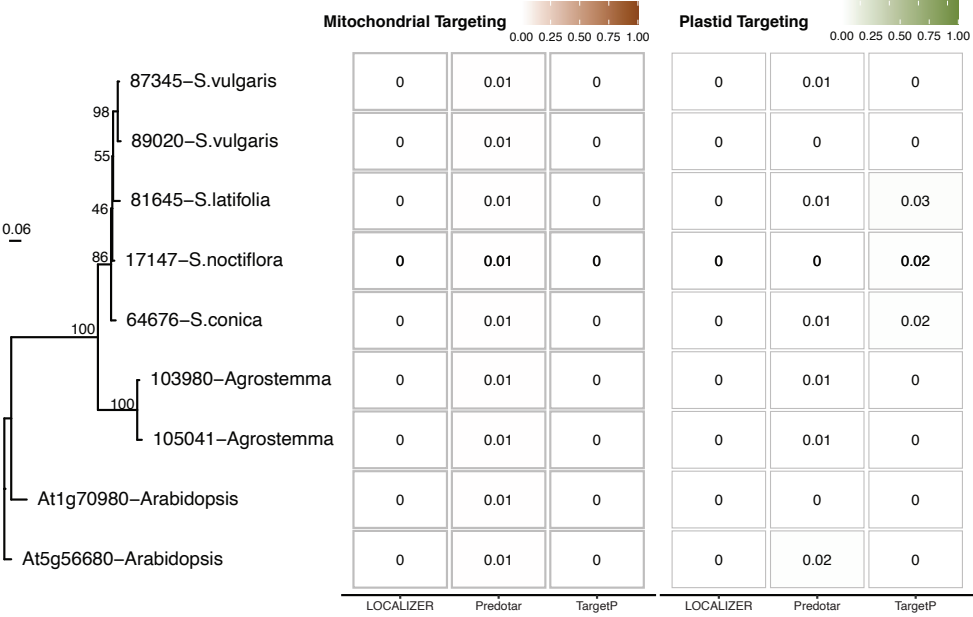

Cytosolic AsnRS

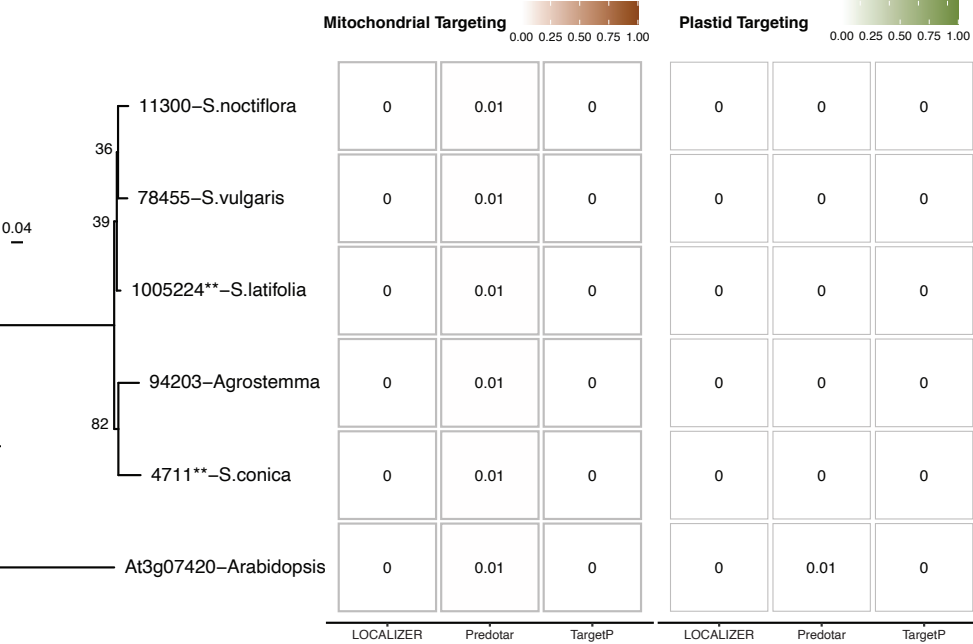

Organellar AsnRS

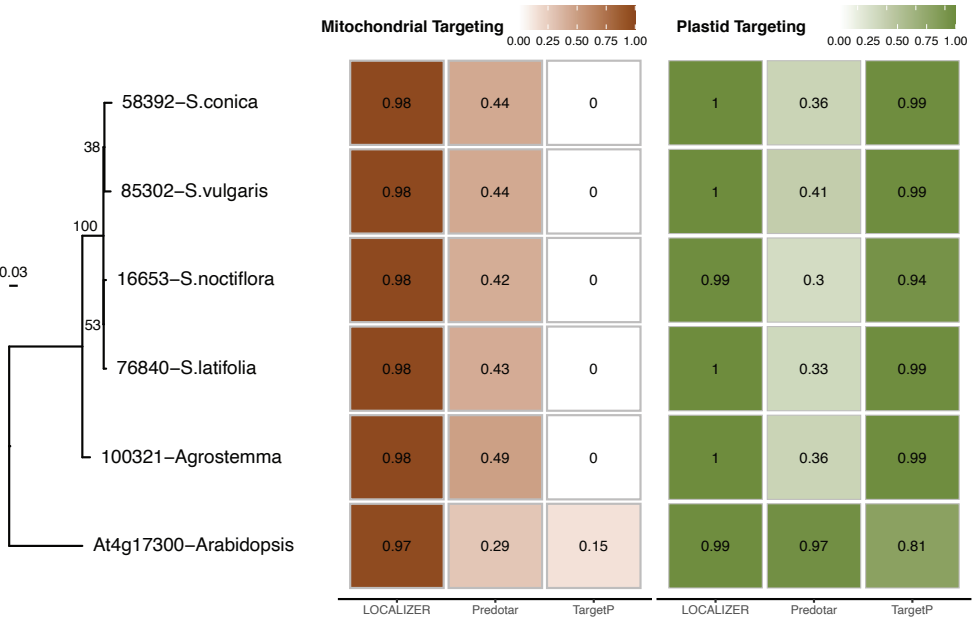

Supplement: msad163_Supplementary_Data [file msad163_supplementary_data.zip › Supp.fig3_AsnRS.pdf]

Cytosolic AspRS

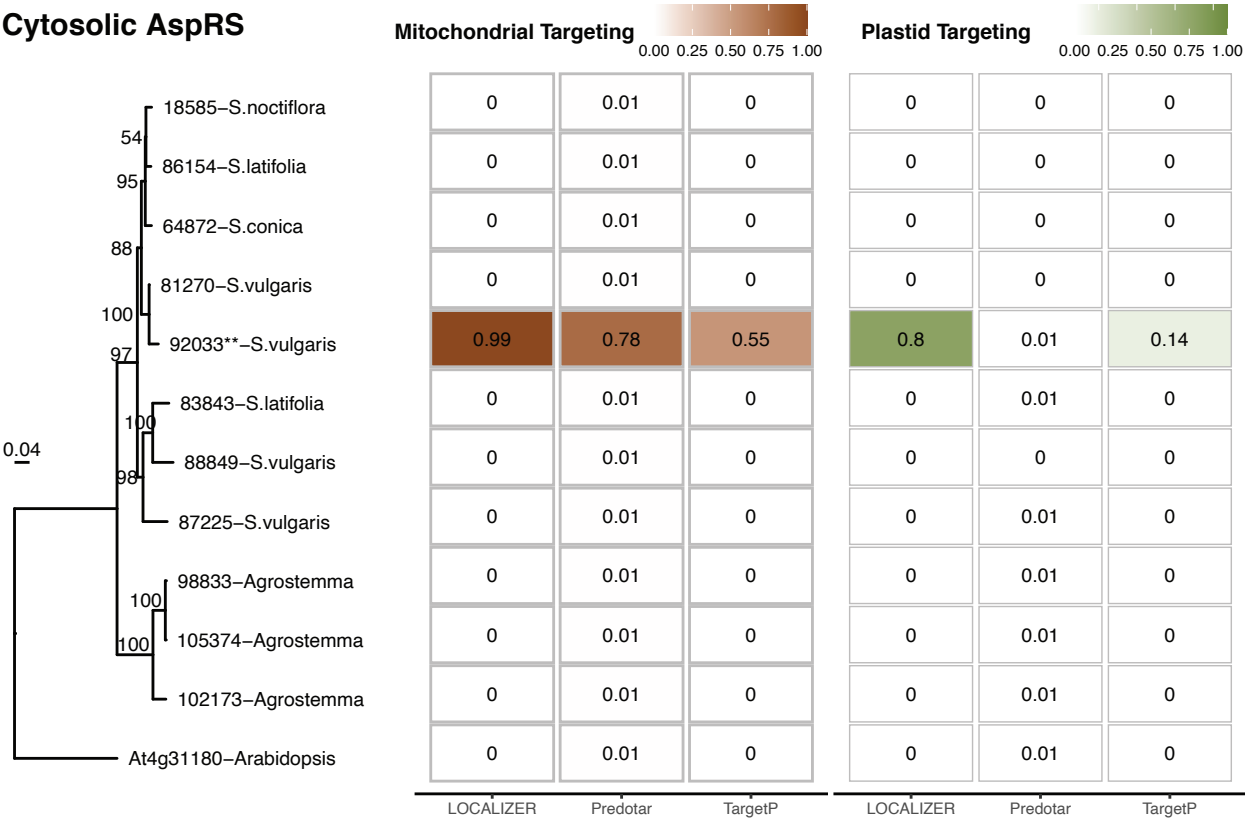

Organellar AspRS

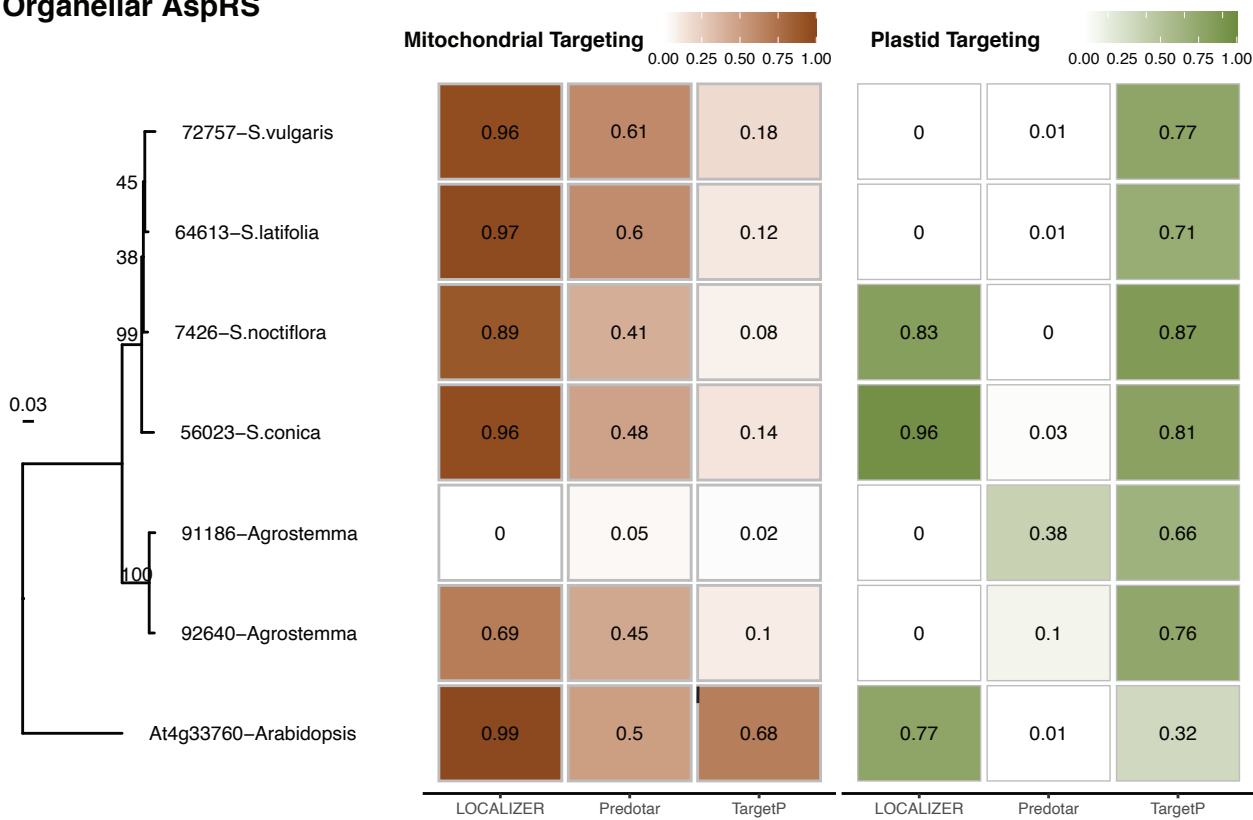

Supplement: msad163_Supplementary_Data [file msad163_supplementary_data.zip › Supp.fig4_AspRS.pdf]

Cytosolic CysRS

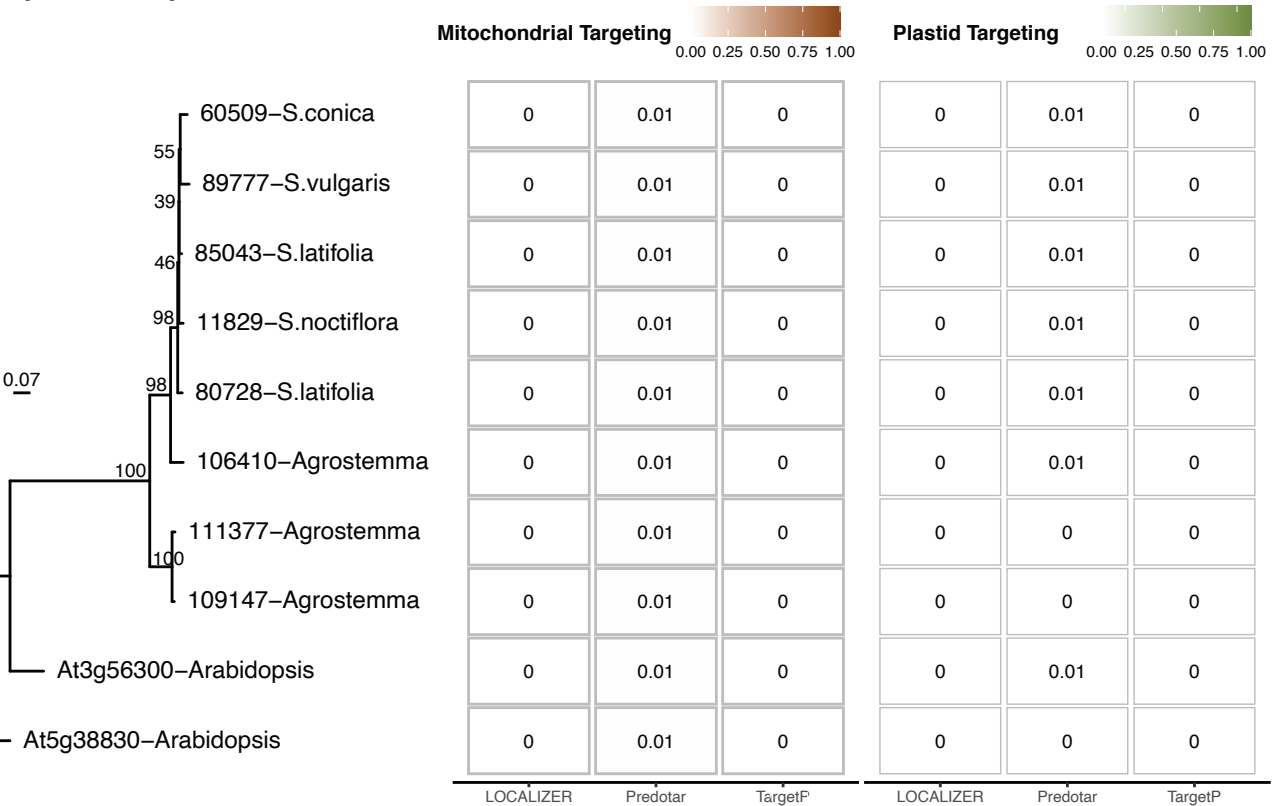

Organellar CysRS

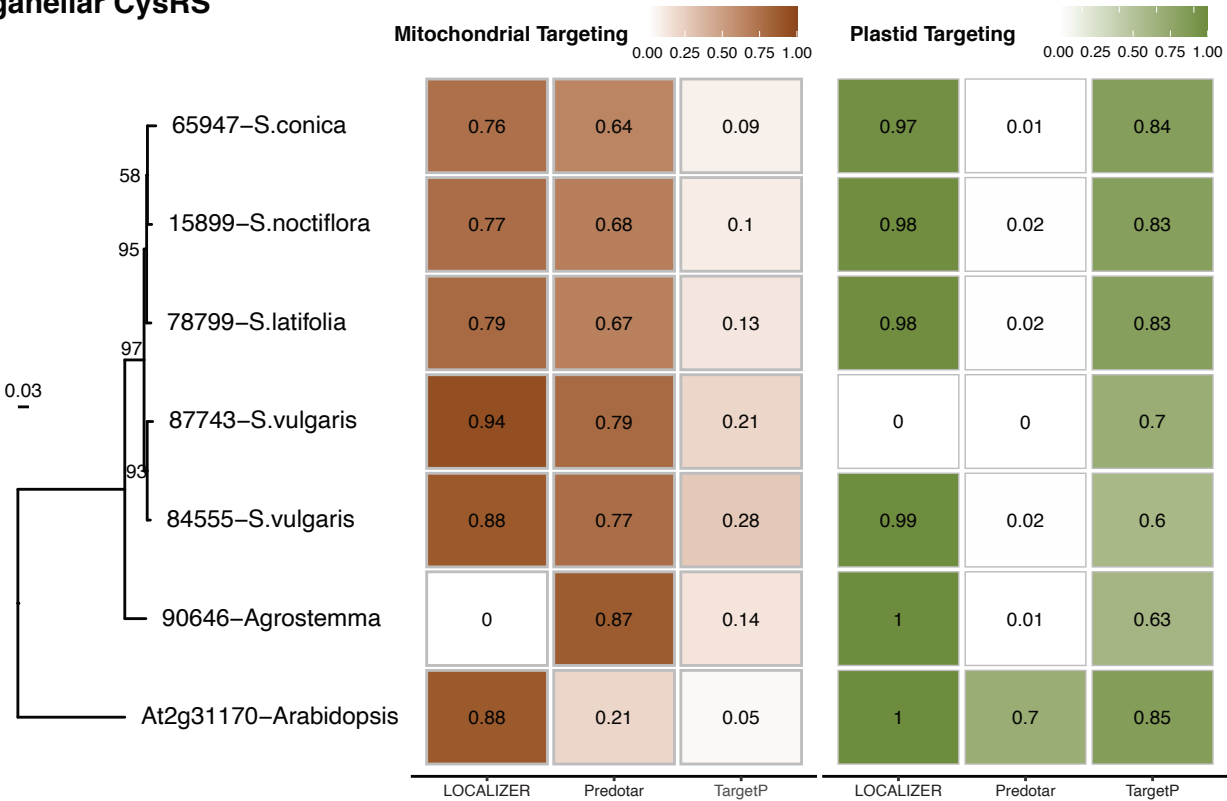

Supplement: msad163_Supplementary_Data [file msad163_supplementary_data.zip › Supp.fig5_CysRS.pdf]

## Cytosolic GluRS

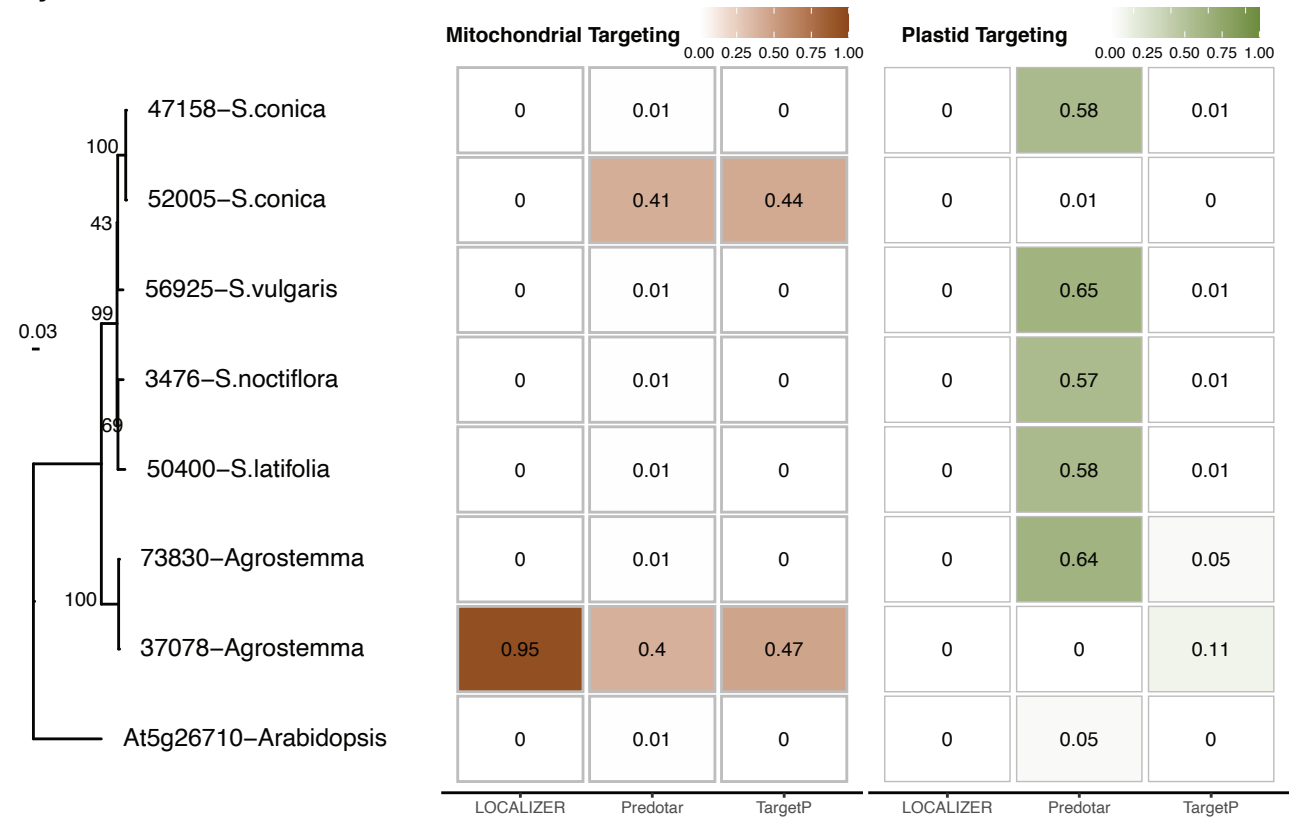

## Organellar GluRS

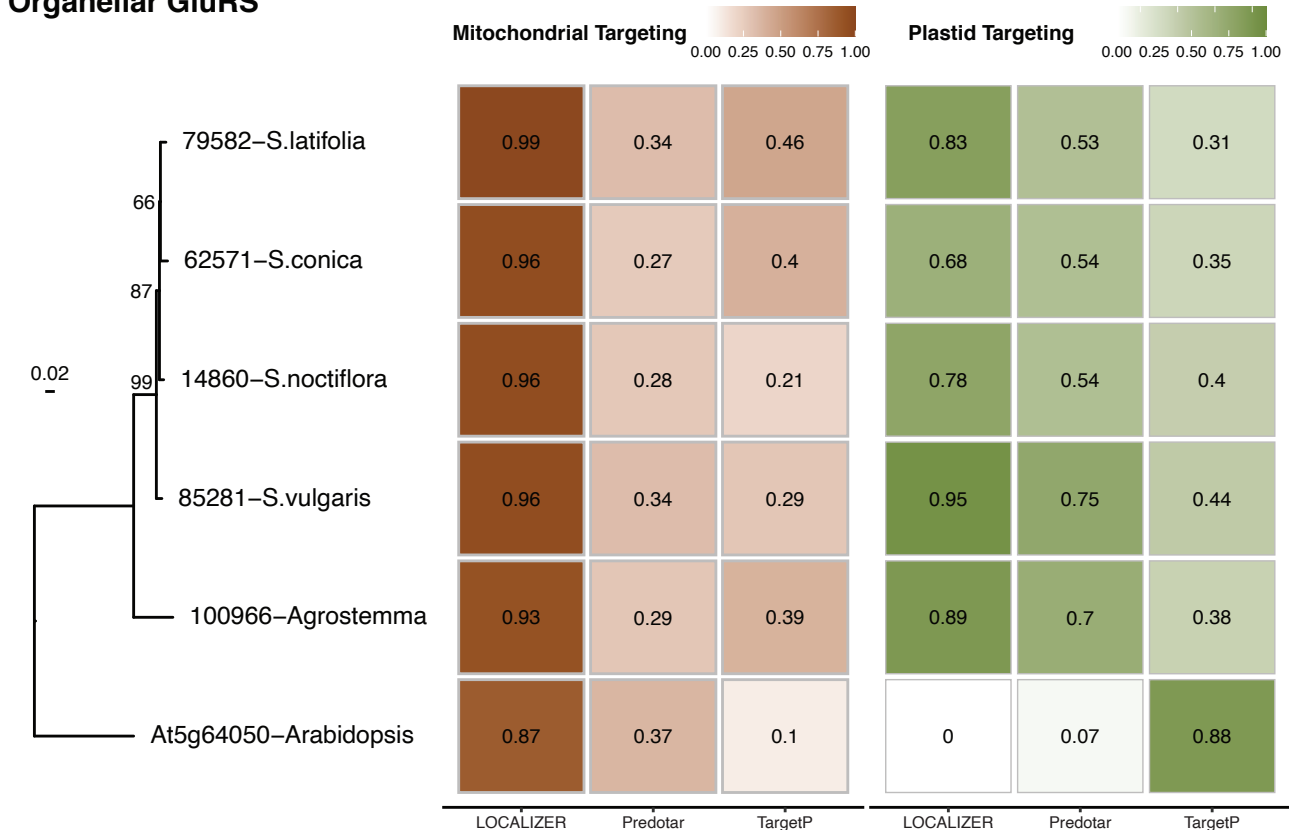

Supplement: msad163_Supplementary_Data [file msad163_supplementary_data.zip › Supp.fig7_GluRS.pdf]

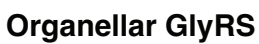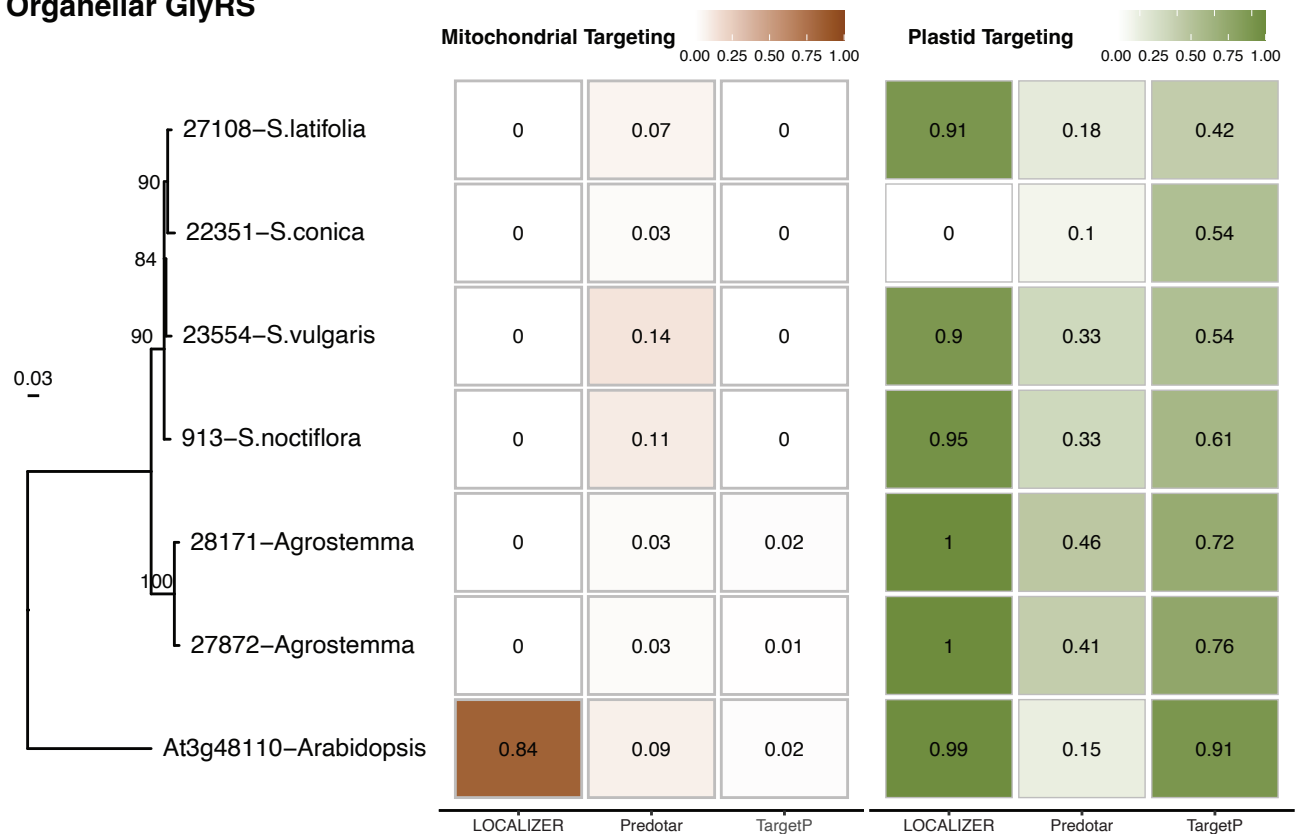

Supplement: msad163_Supplementary_Data [file msad163_supplementary_data.zip › Supp.fig8_GlyRS.pdf]

Cytosolic HisRS

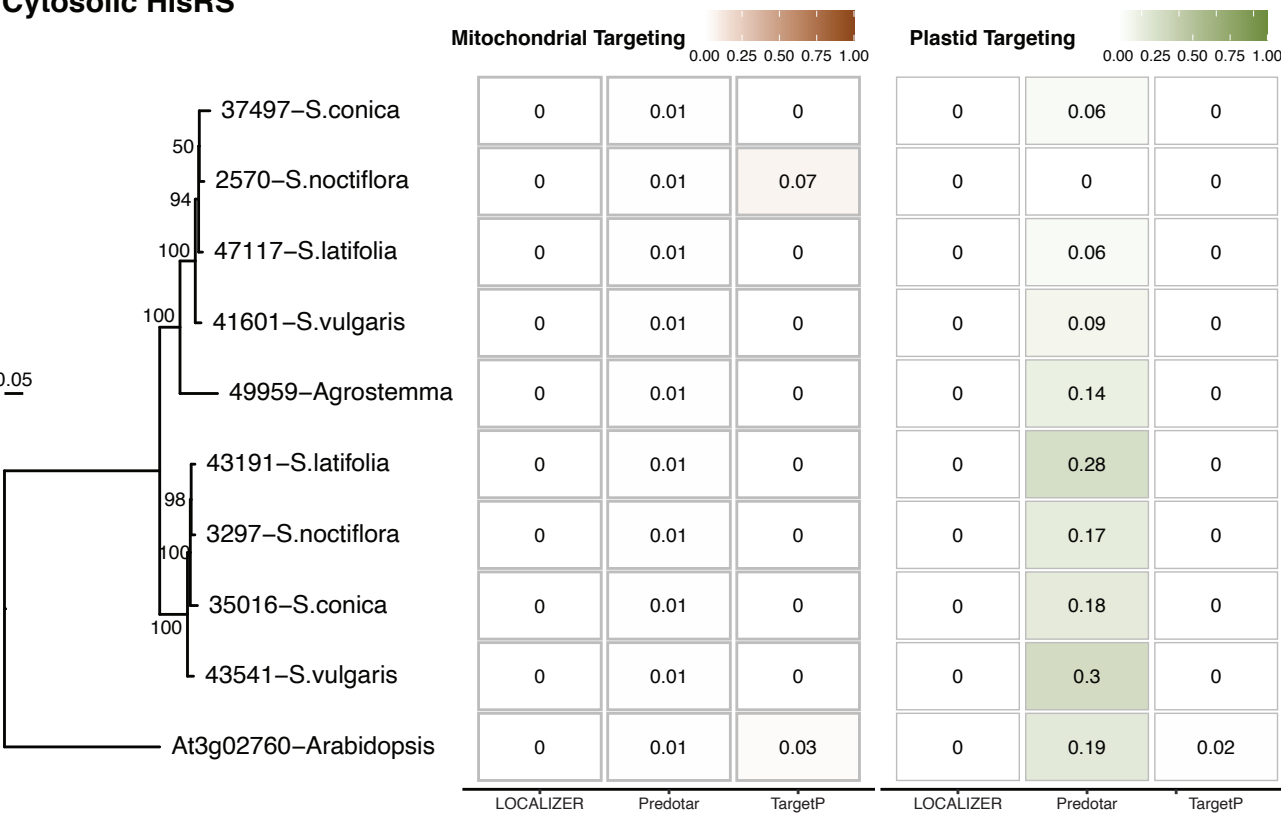

Organellar HisRS

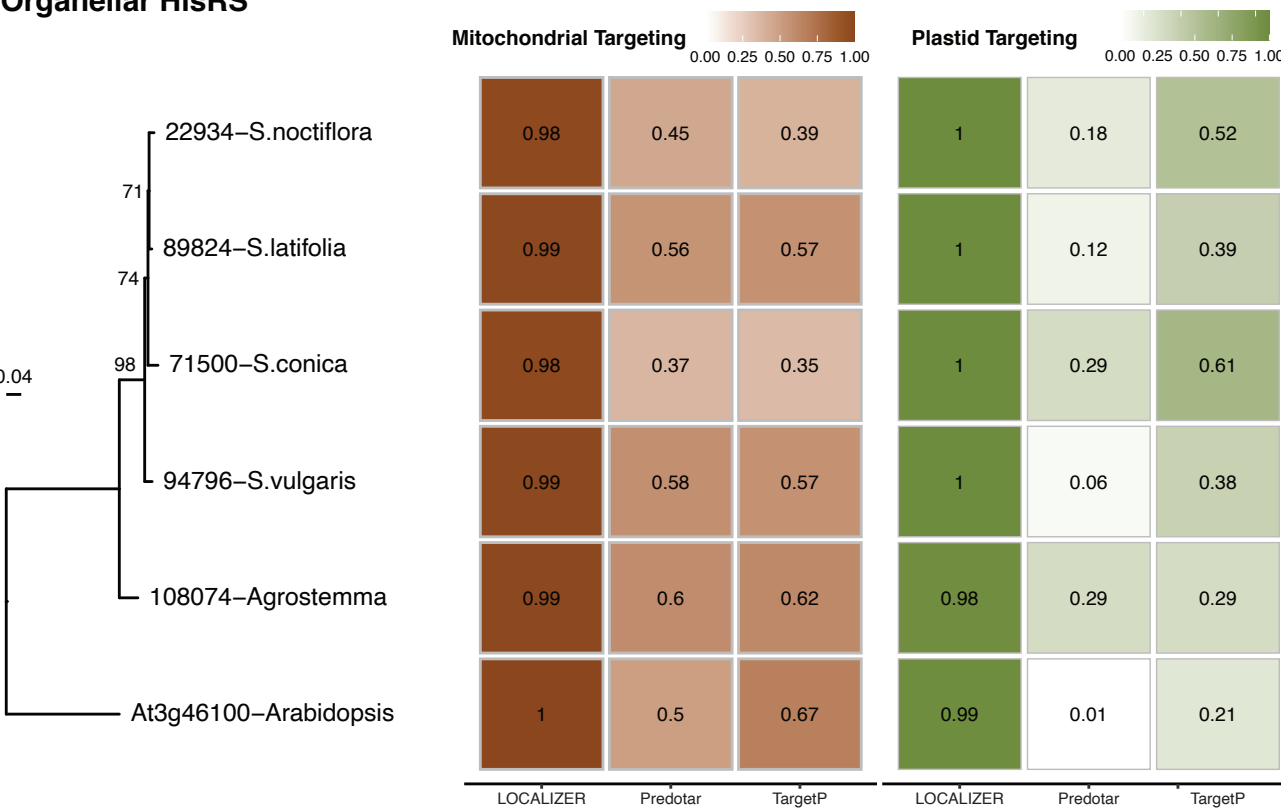

Supplement: msad163_Supplementary_Data [file msad163_supplementary_data.zip › Supp.fig9_HisRS.pdf]

## Cytosolic IleRS

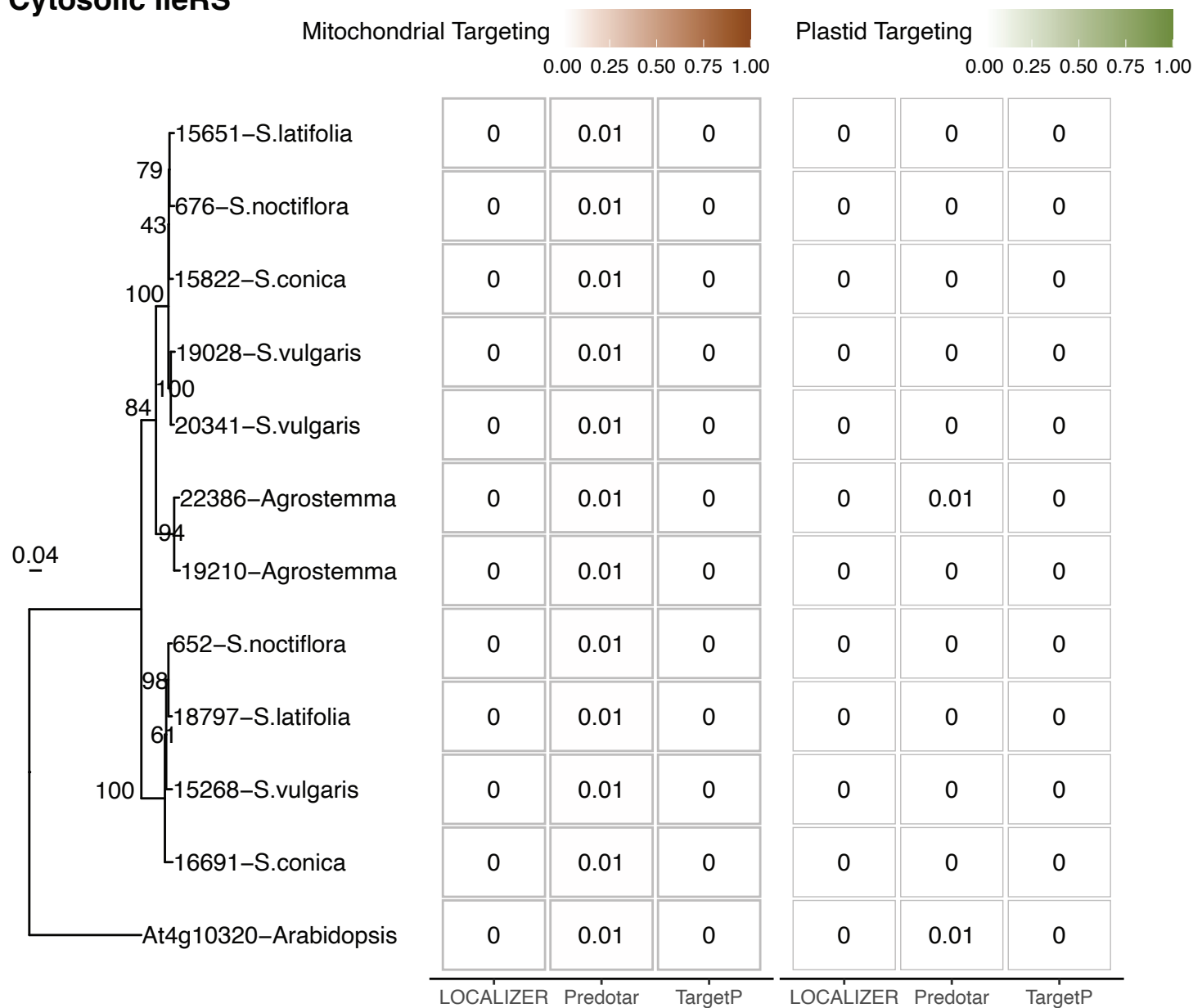

## Organellar IleRS

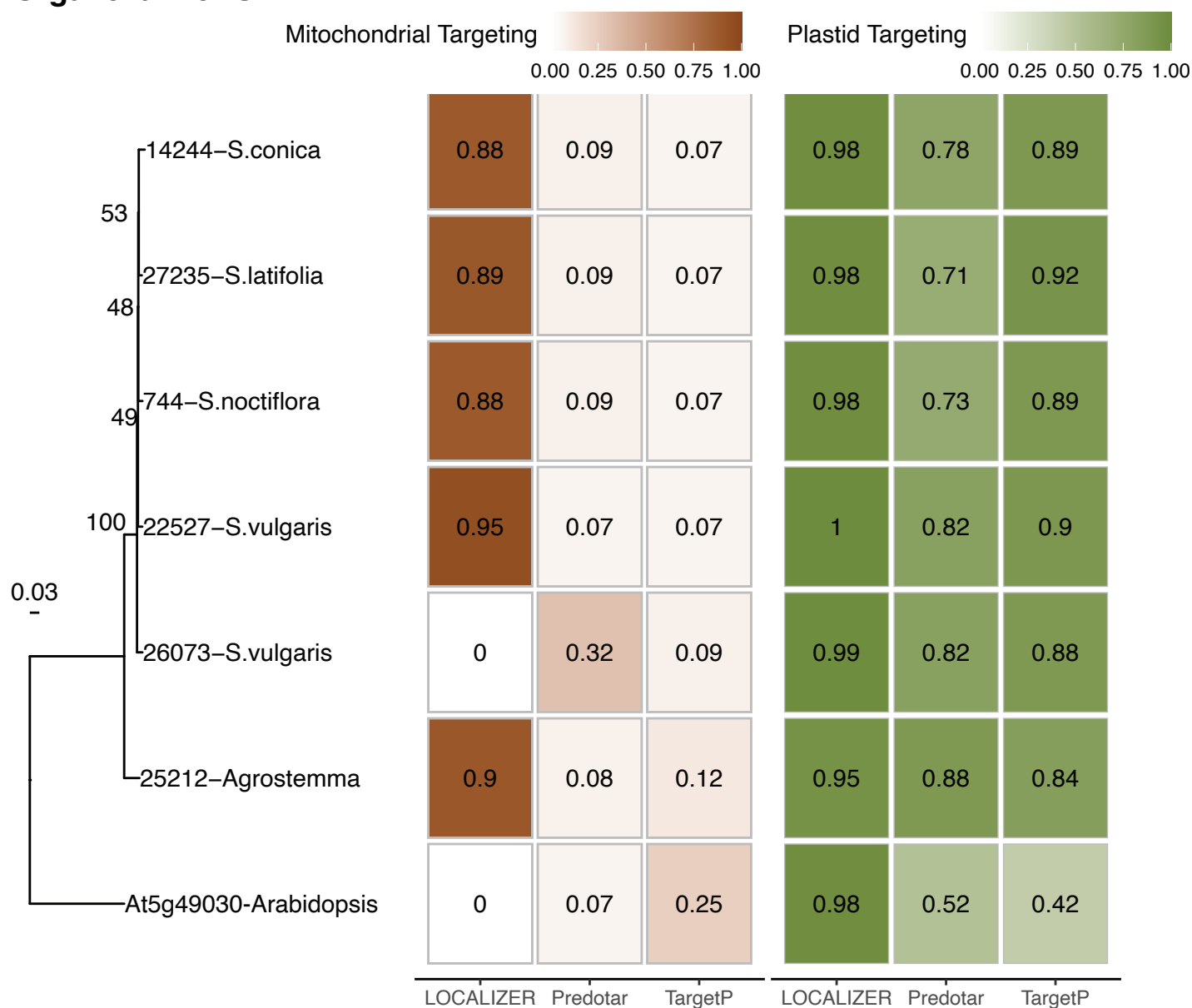

Supplement: msad163_Supplementary_Data [file msad163_supplementary_data.zip › Supp.fig10_IleRS.pdf]

Cytosolic/Organellar LeuRS

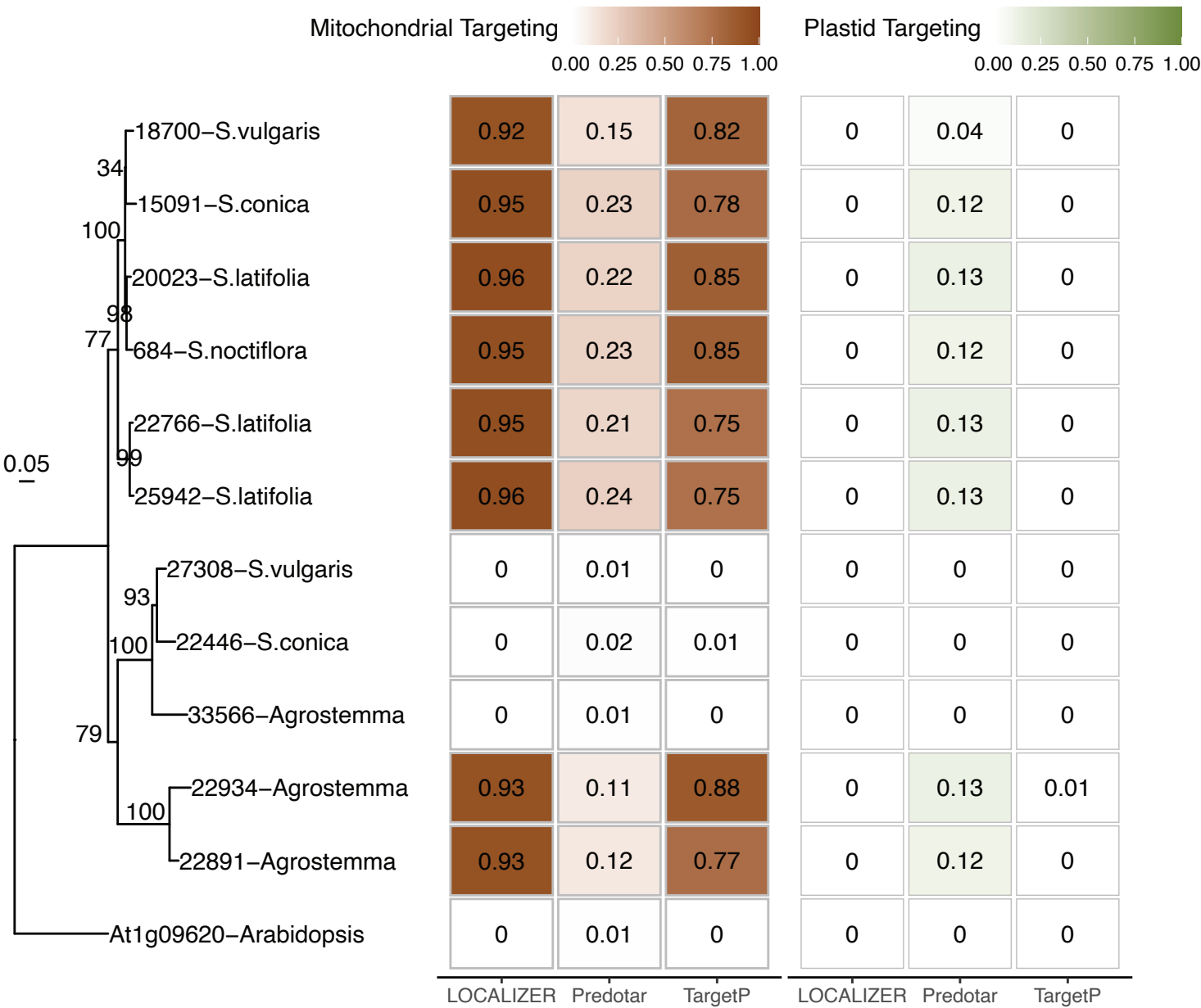

Supplement: msad163_Supplementary_Data [file msad163_supplementary_data.zip › Supp.fig11_LeuRS.pdf]

Cytosolic LysRS

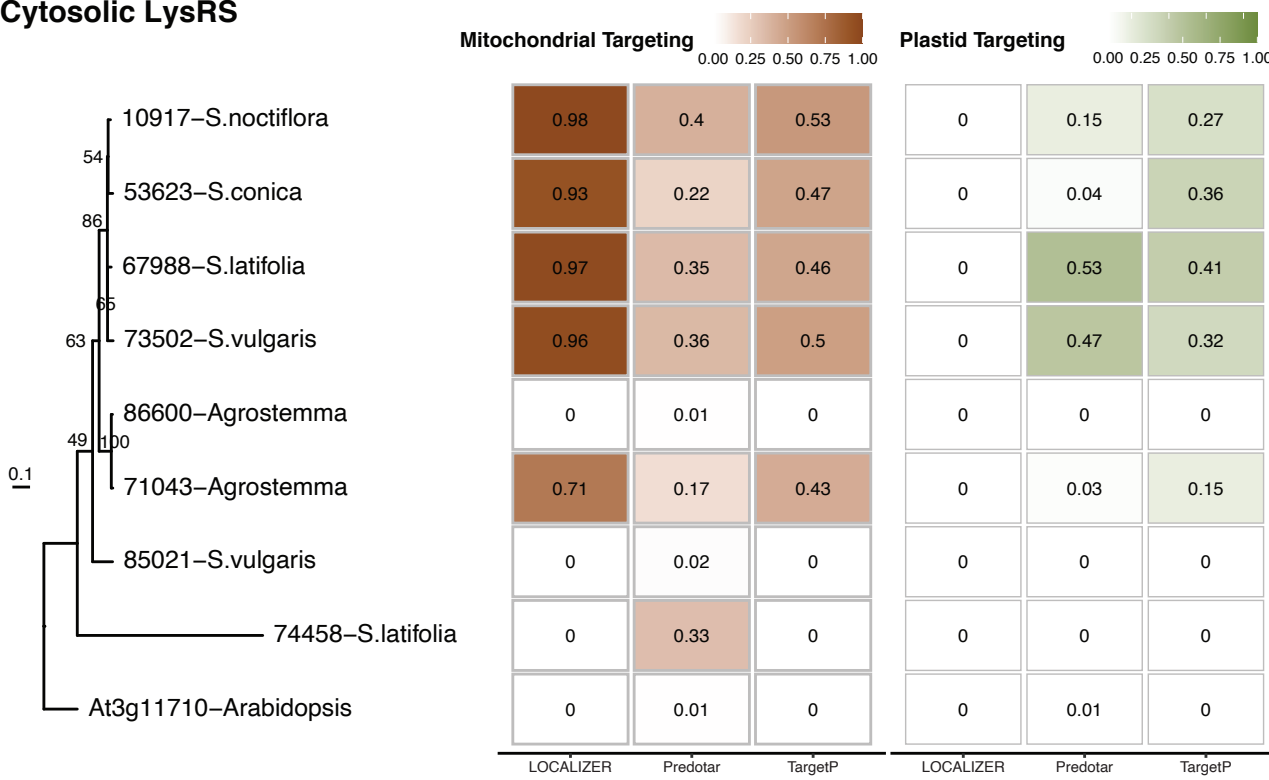

Organelar LysRS

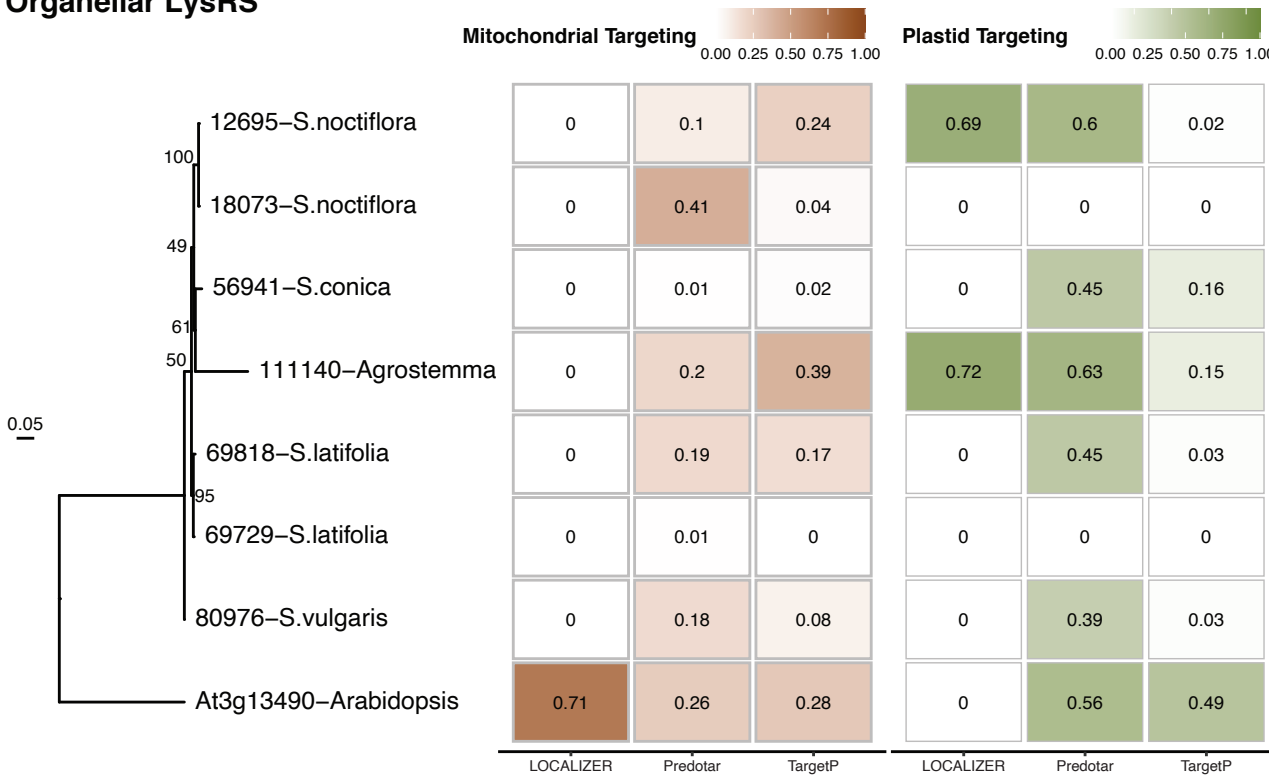

Supplement: msad163_Supplementary_Data [file msad163_supplementary_data.zip › Supp.fig12_LysRS.pdf]

## Cytosolic MetRS 1

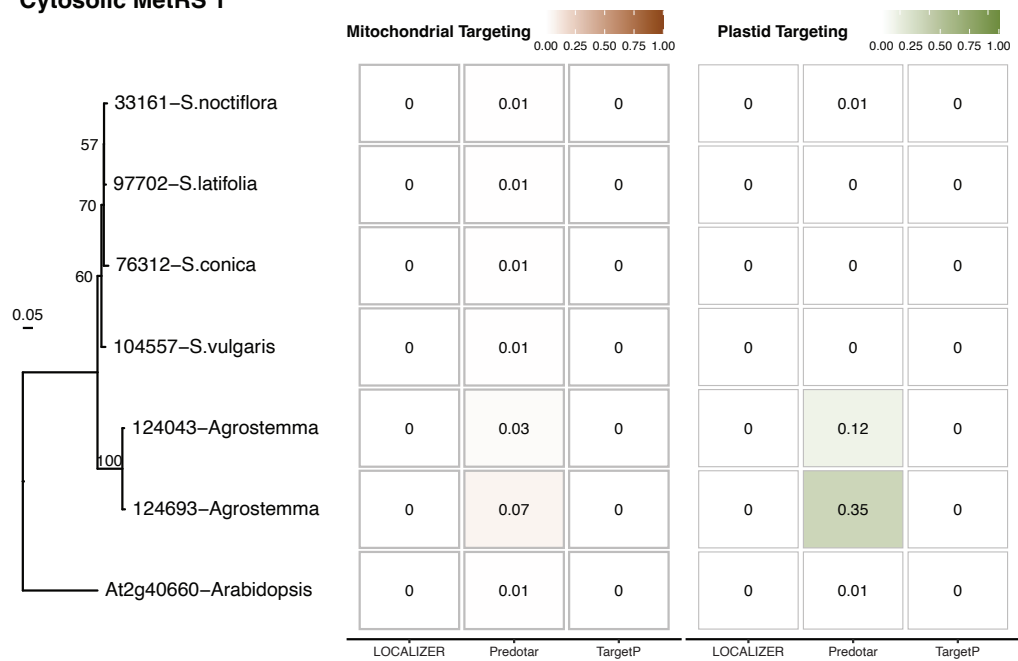

## Cytosolic MetRS 2

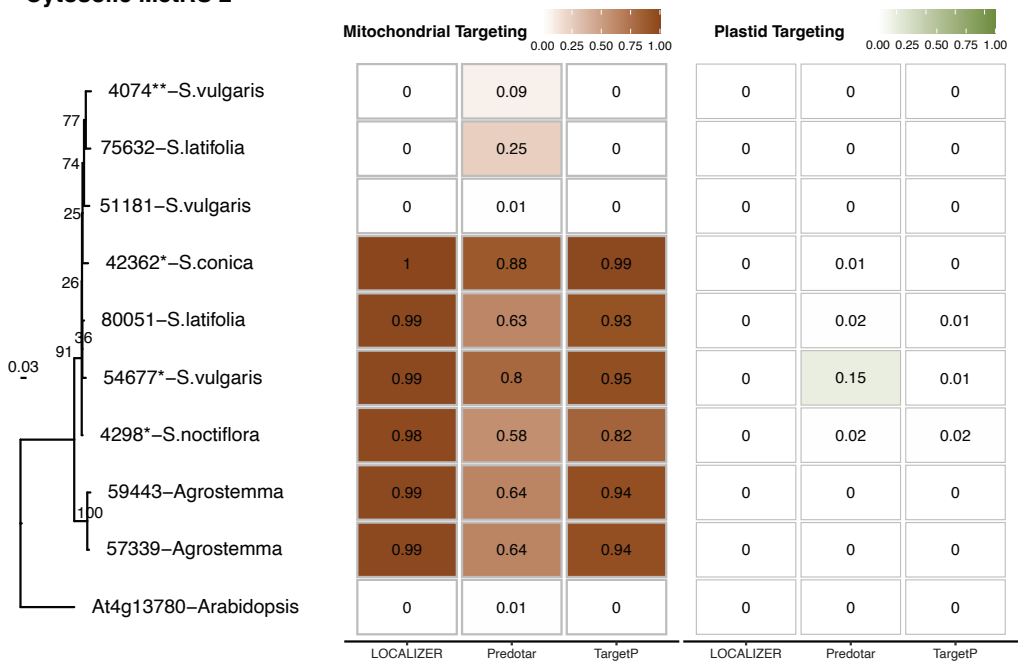

## Organellar MetRS

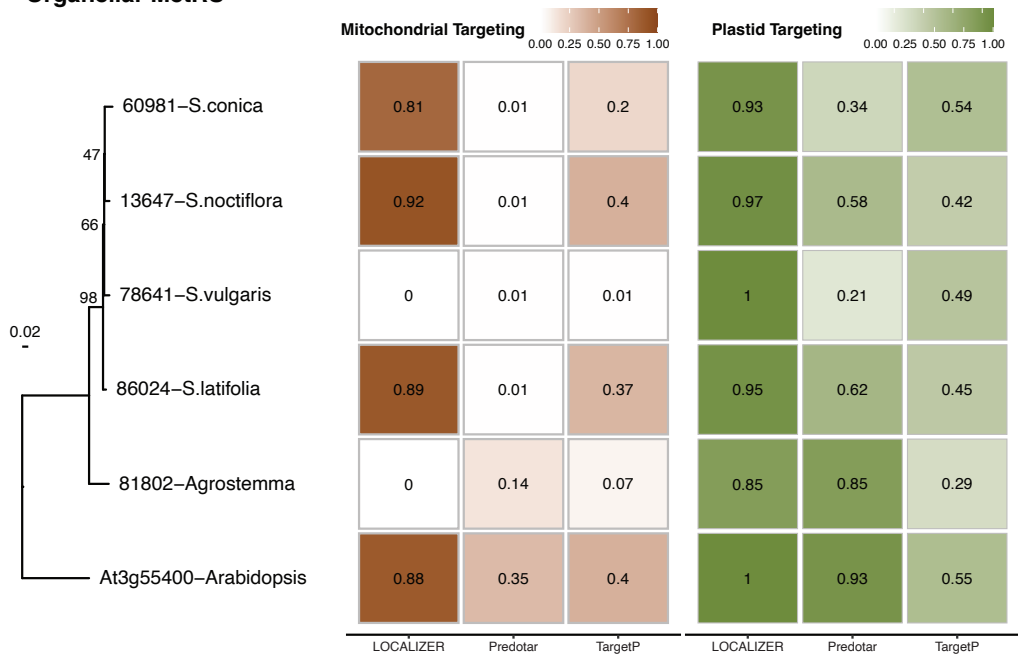

Supplement: msad163_Supplementary_Data [file msad163_supplementary_data.zip › Supp.fig13_Met.pdf]

Cytosolic PheRS

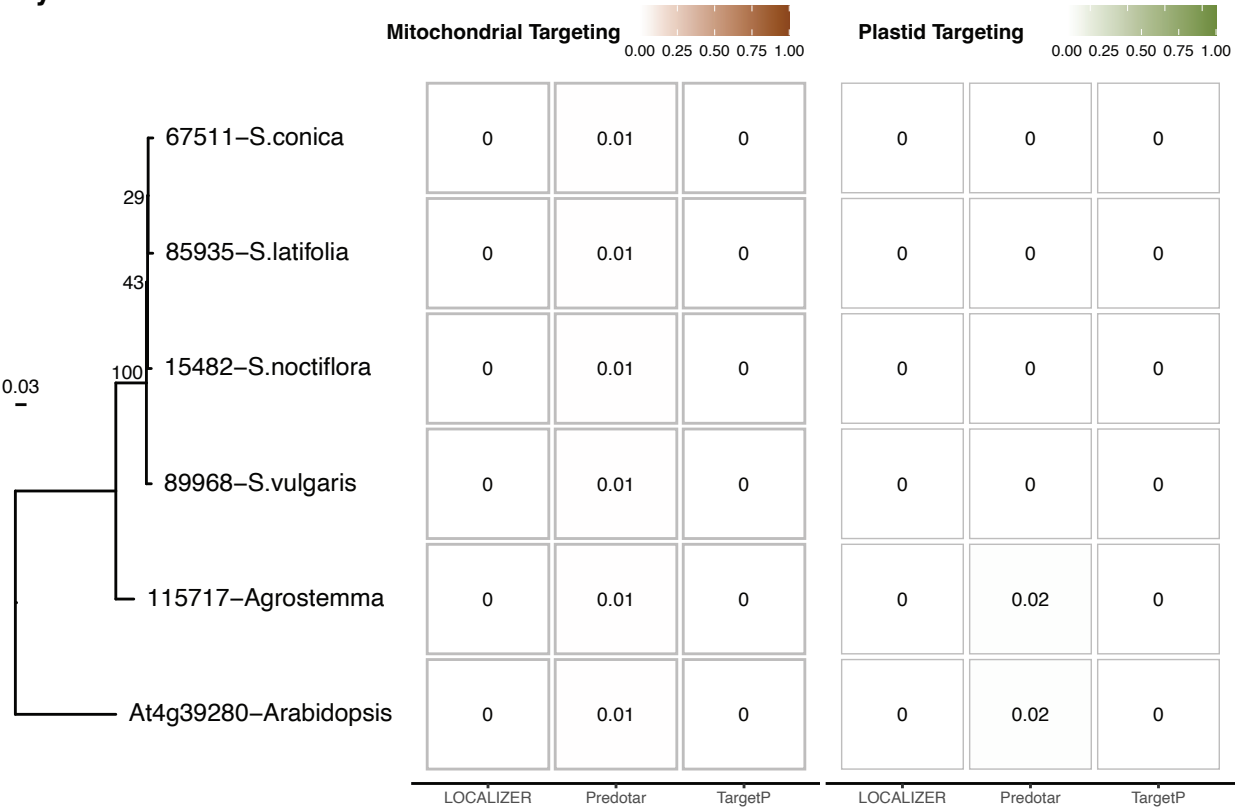

Cytosolic PheRS β-subunit

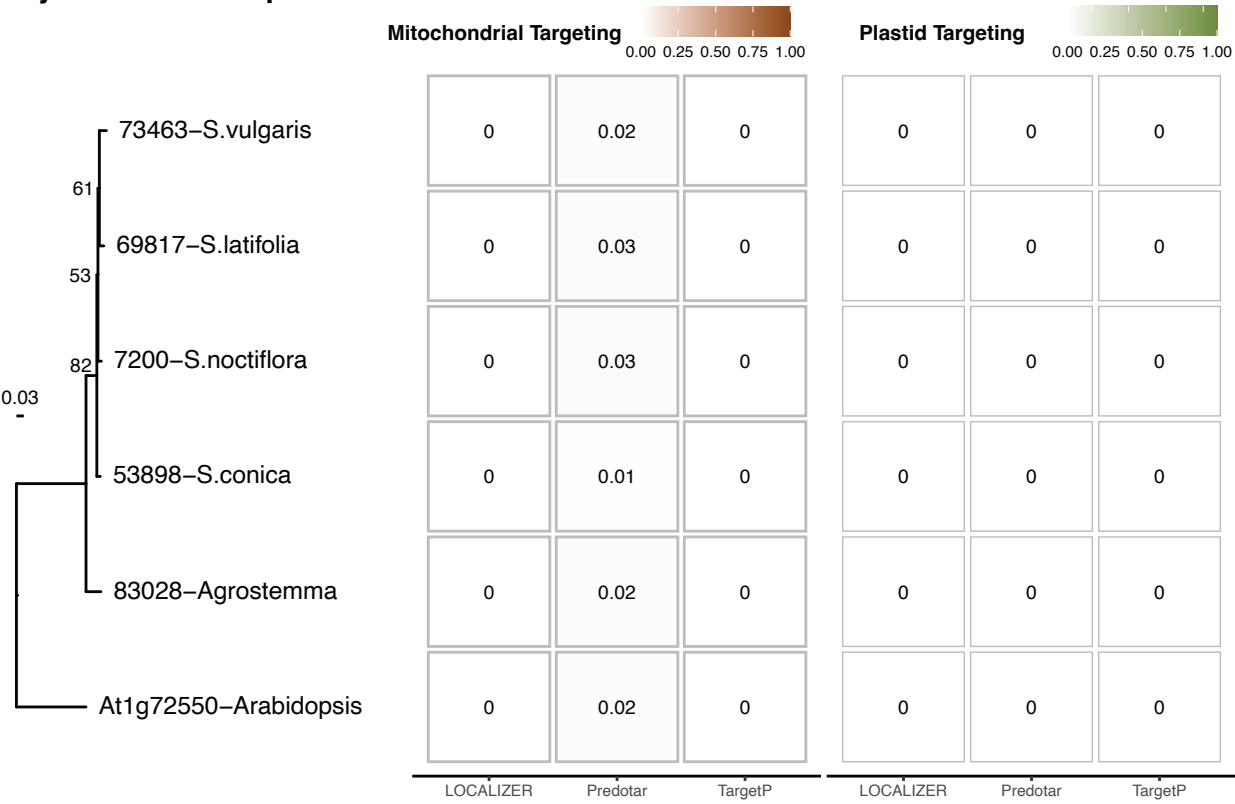

Organellar PheRS

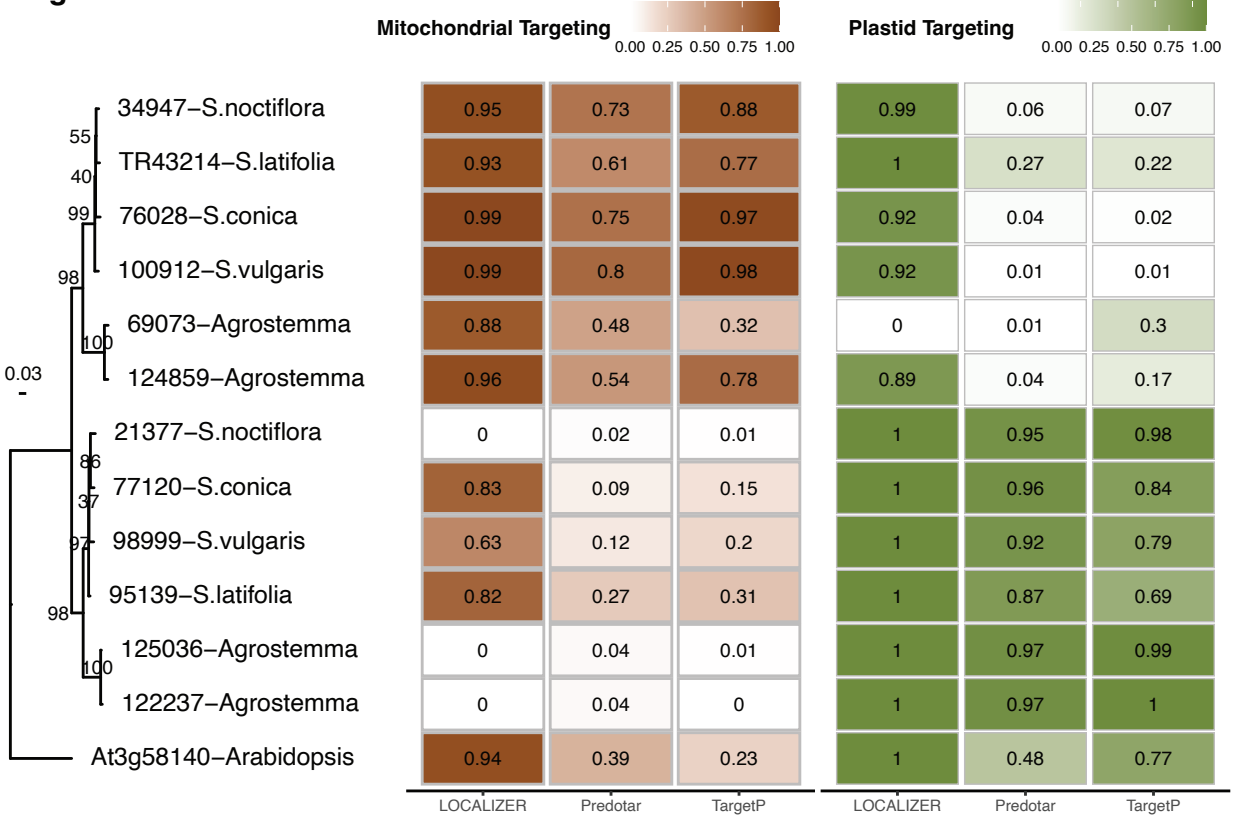

Supplement: msad163_Supplementary_Data [file msad163_supplementary_data.zip › Supp.fig14_PheRS.pdf]

# Cytosolic ProRS

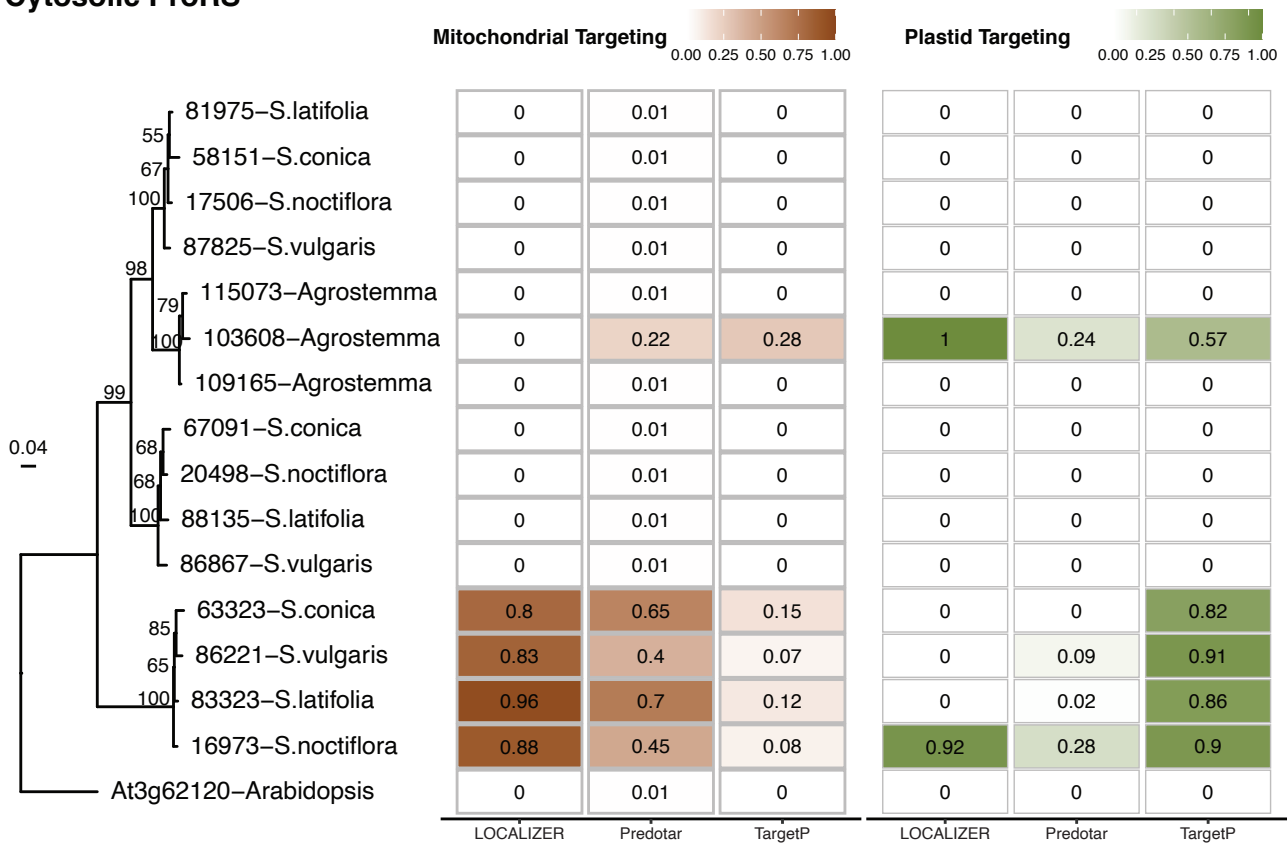

# Organellar ProRS

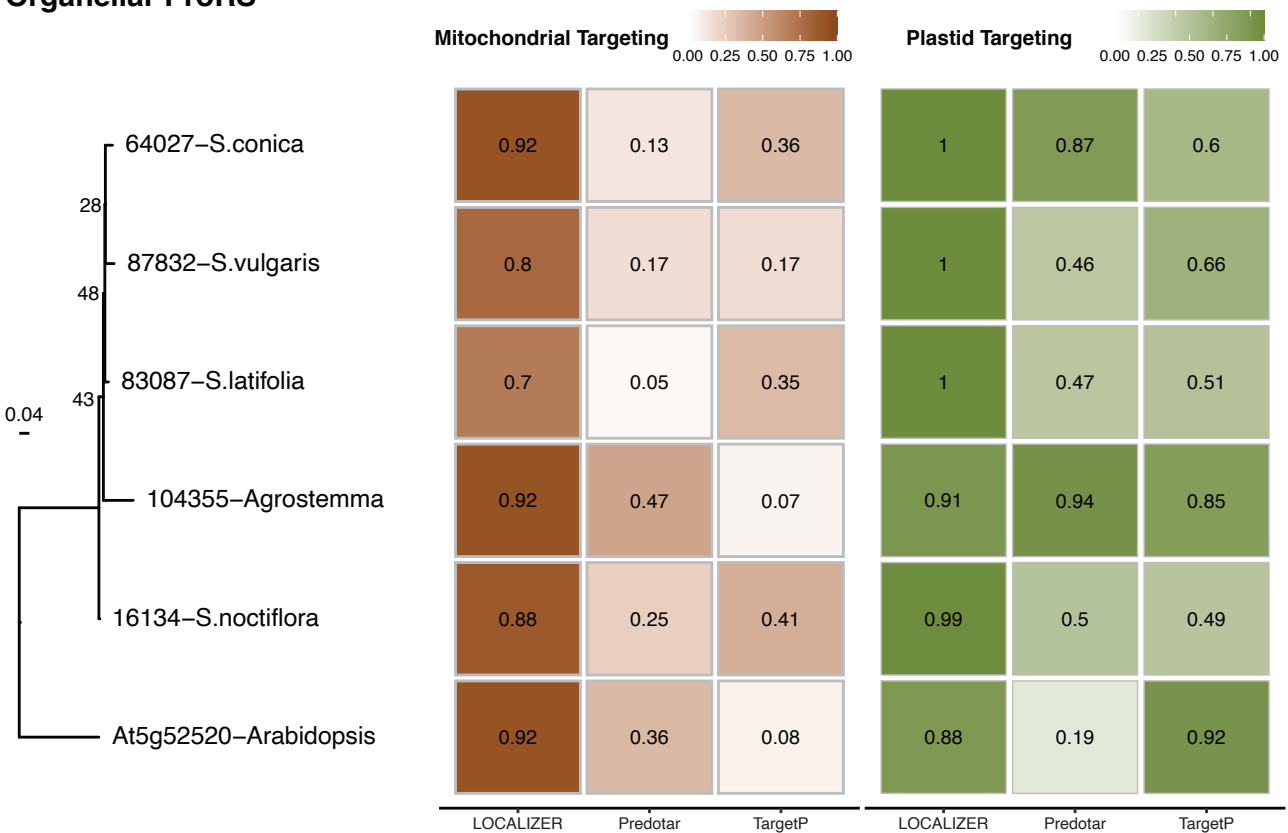

Supplement: msad163_Supplementary_Data [file msad163_supplementary_data.zip › Supp.fig15_ProRS.pdf]

Cytosolic SerRS

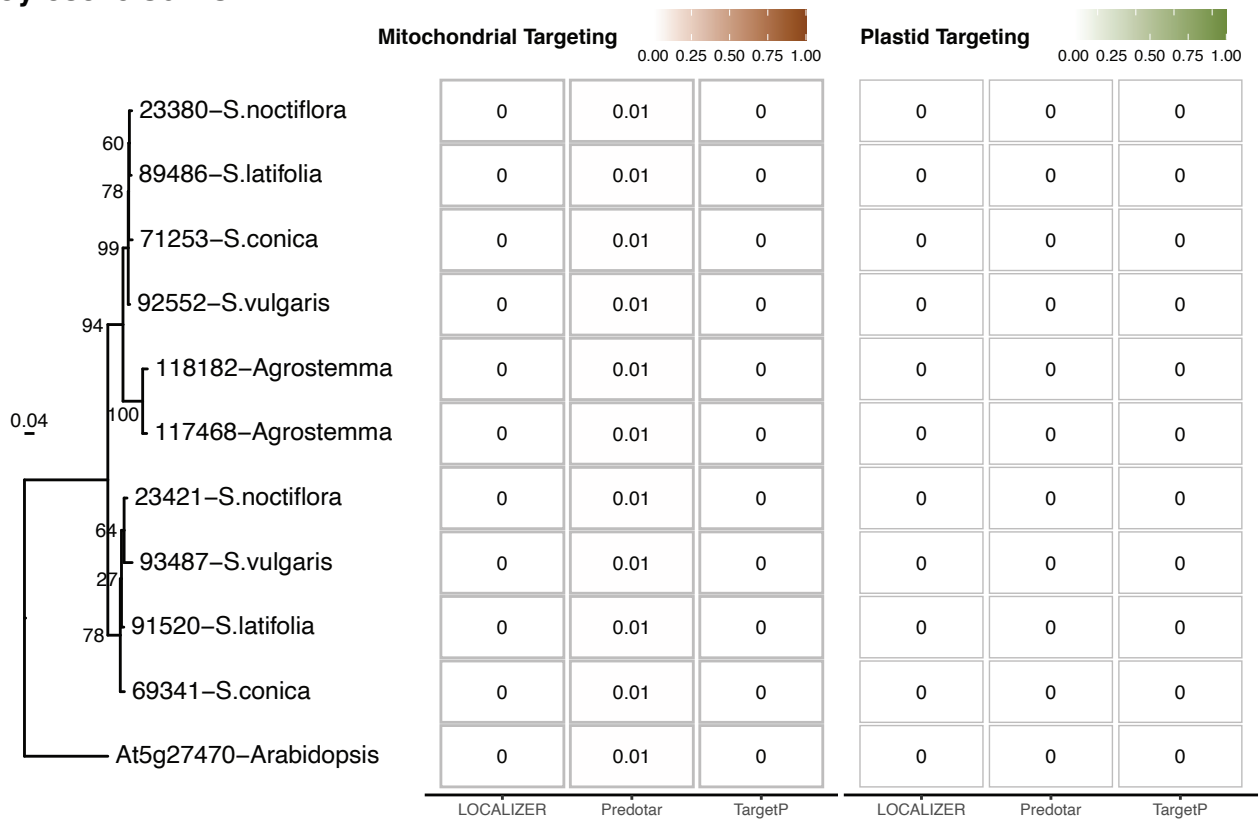

Organellar SerRS

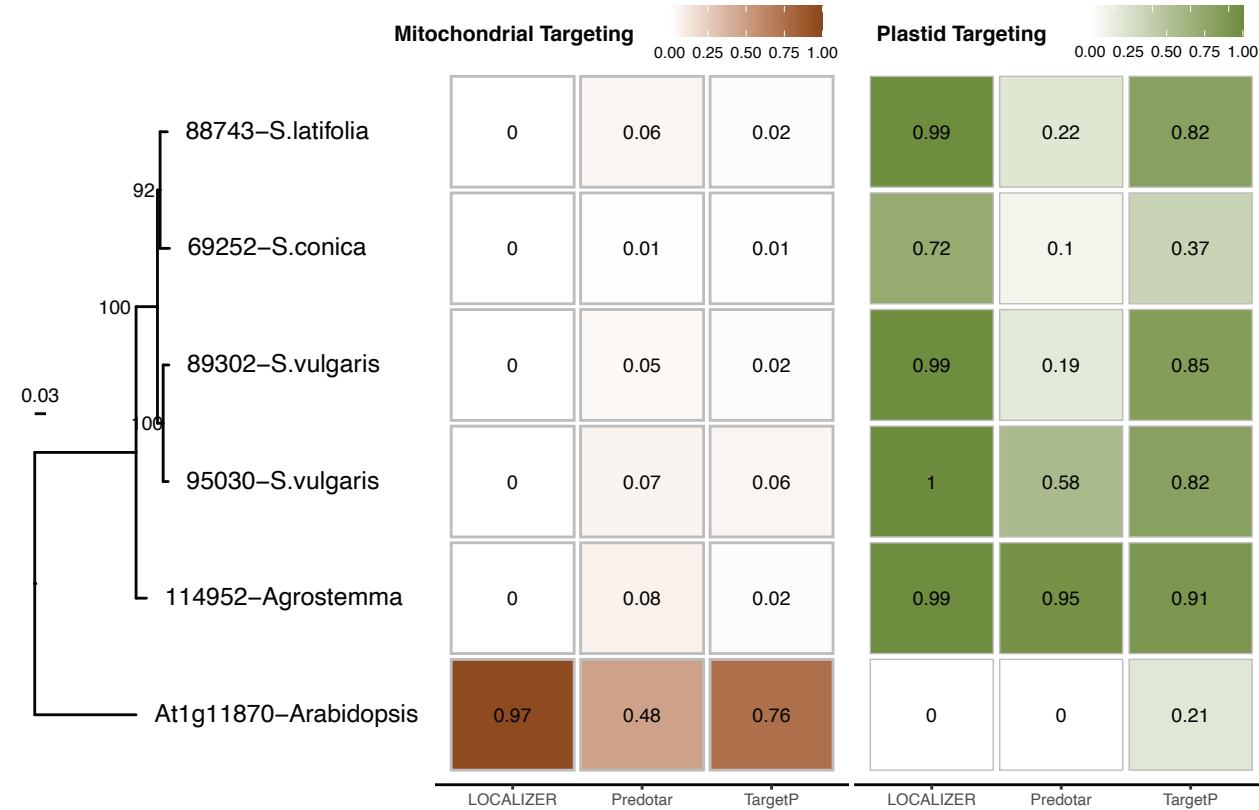

Supplement: msad163_Supplementary_Data [file msad163_supplementary_data.zip › Supp.fig16_SerRS.pdf]

Cytosolic/Organellar ThrRS

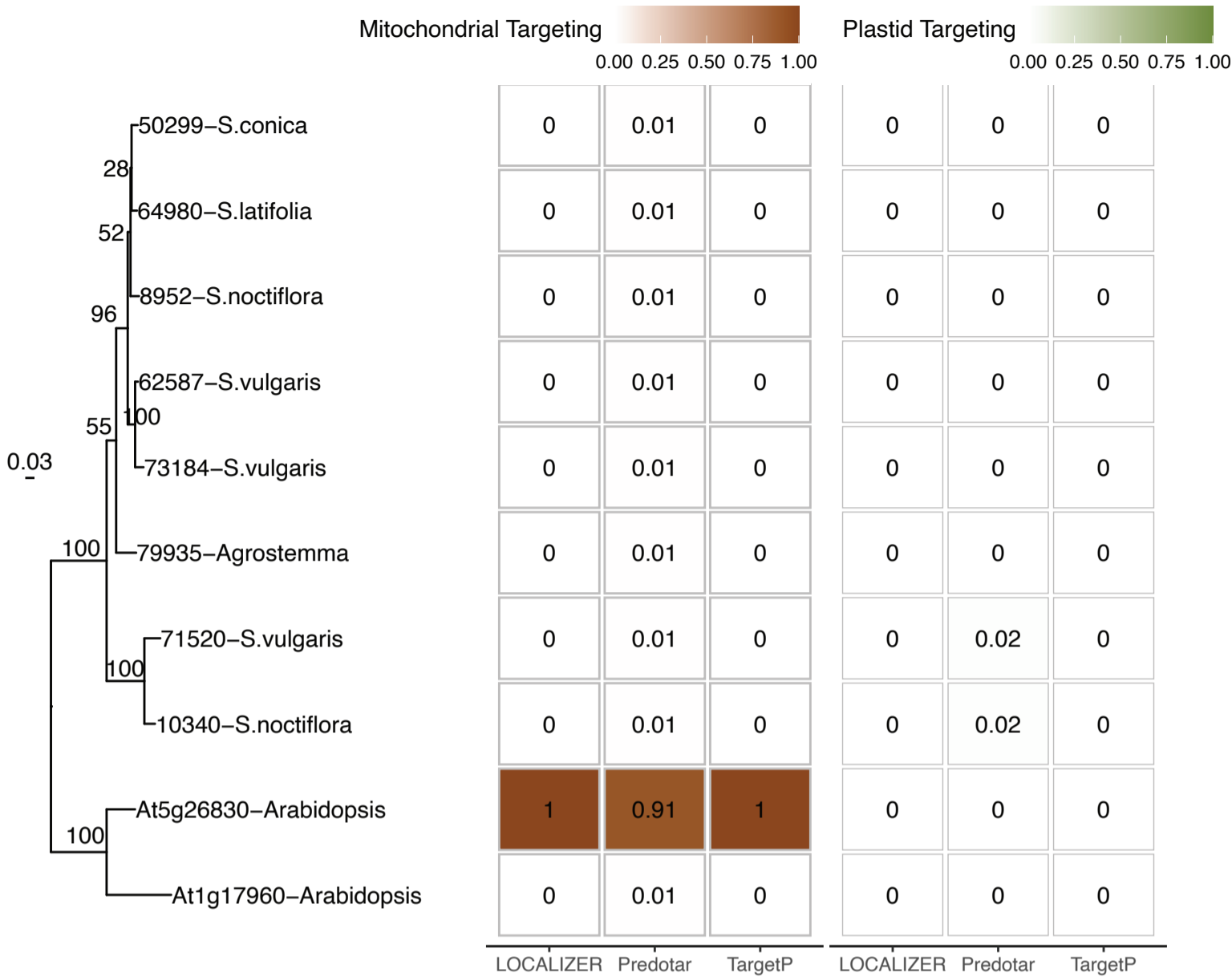

Organellar ThrRS

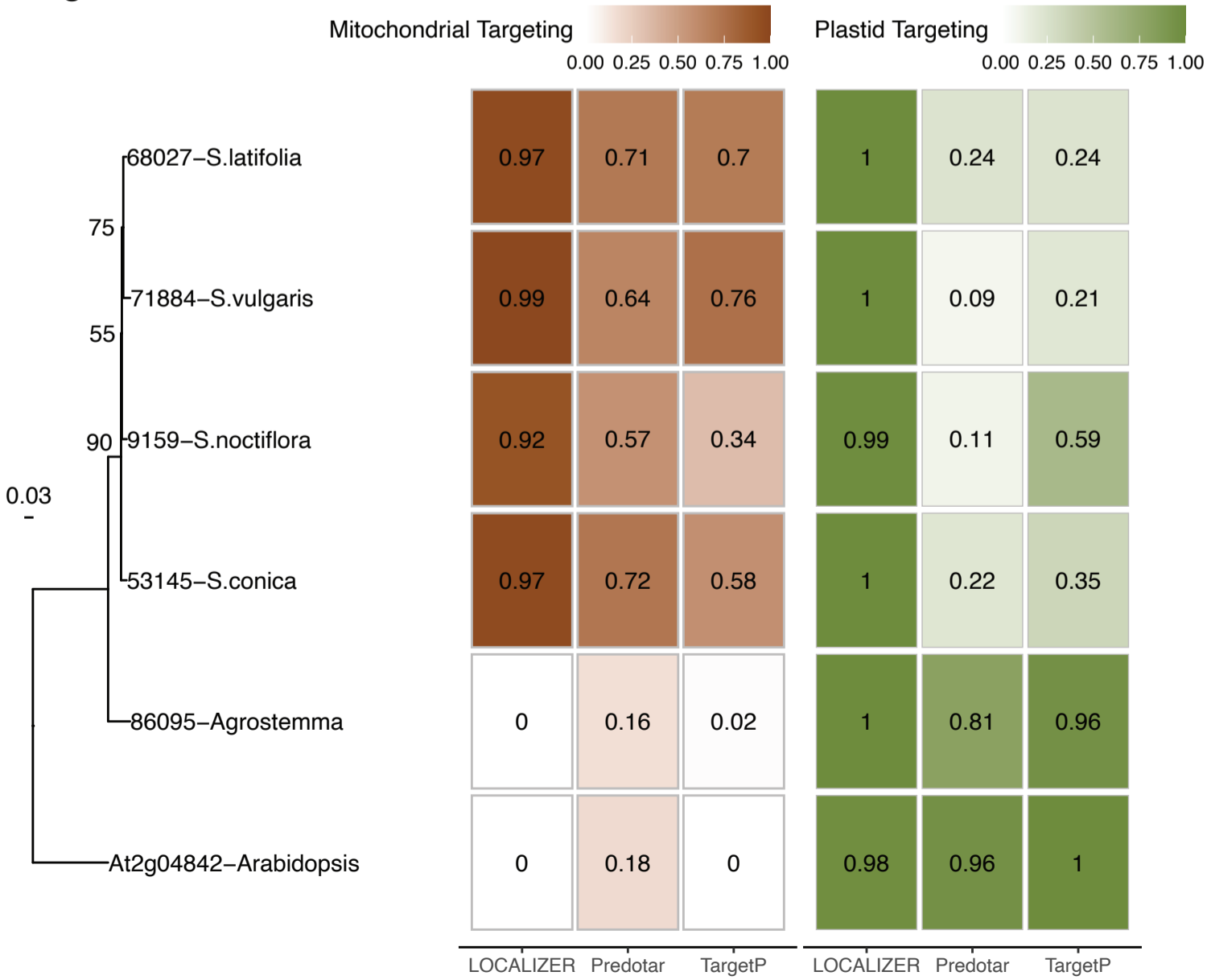

Supplement: msad163_Supplementary_Data [file msad163_supplementary_data.zip › Supp.fig17_ThrRS.pdf]

Cytosolic TrpRS

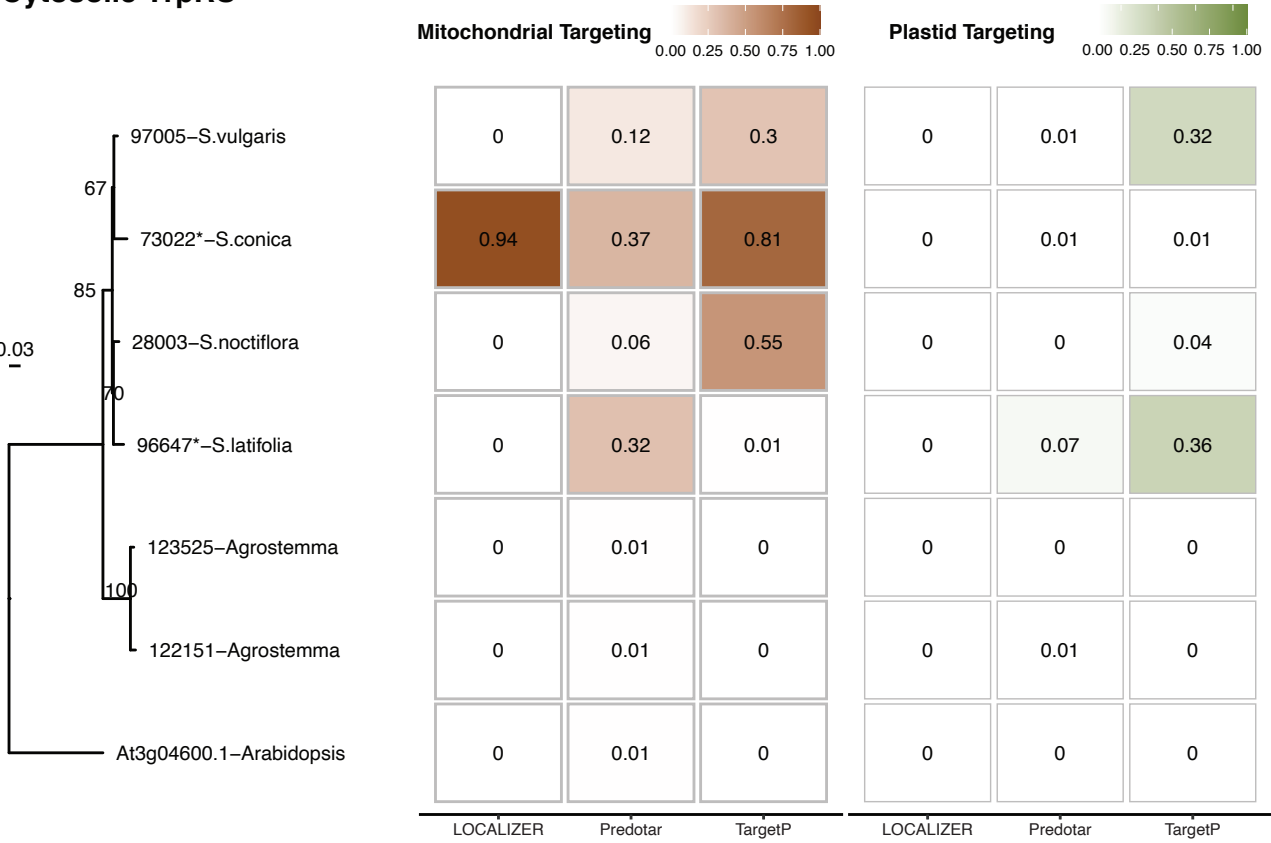

Organelar TrpRS

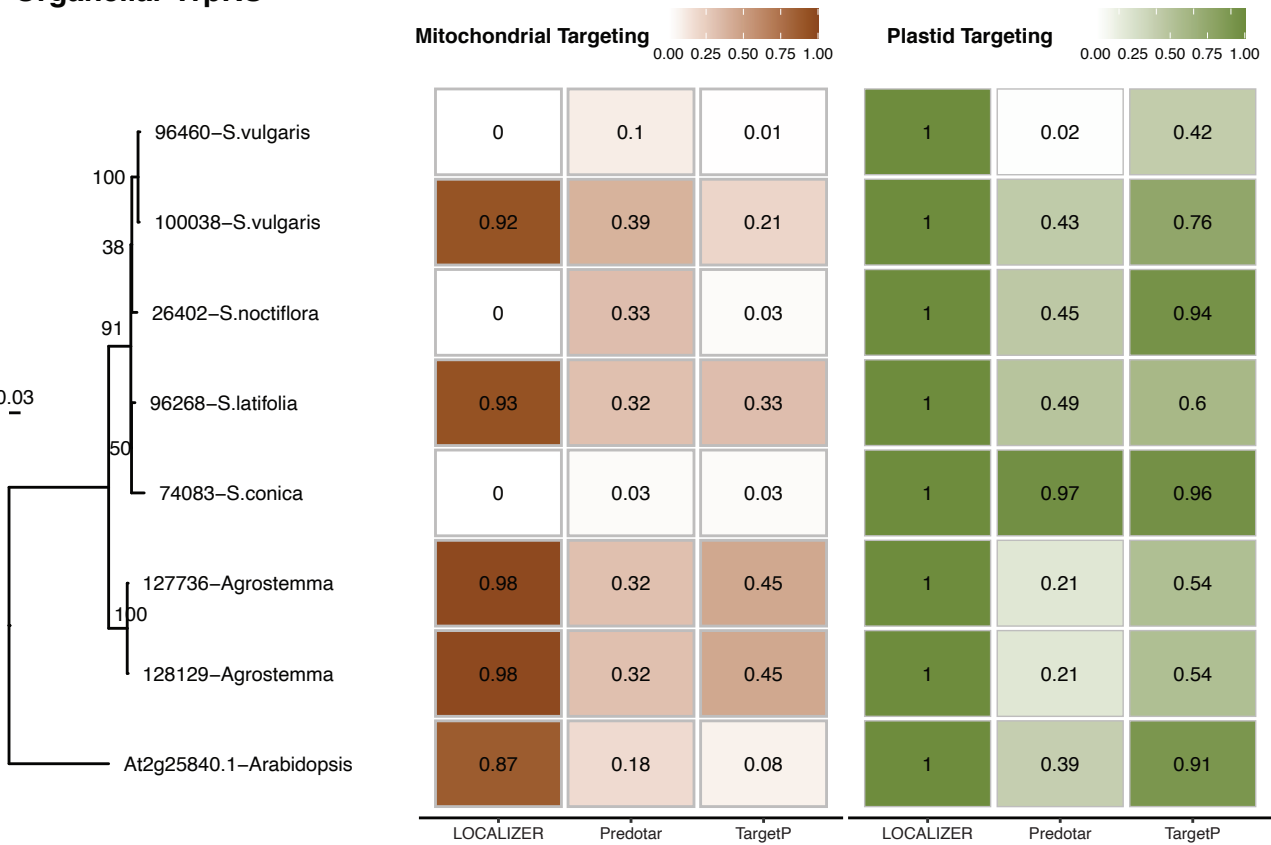

Supplement: msad163_Supplementary_Data [file msad163_supplementary_data.zip › Supp.fig18_TrpRS.pdf]

Cytosolic TyrRS

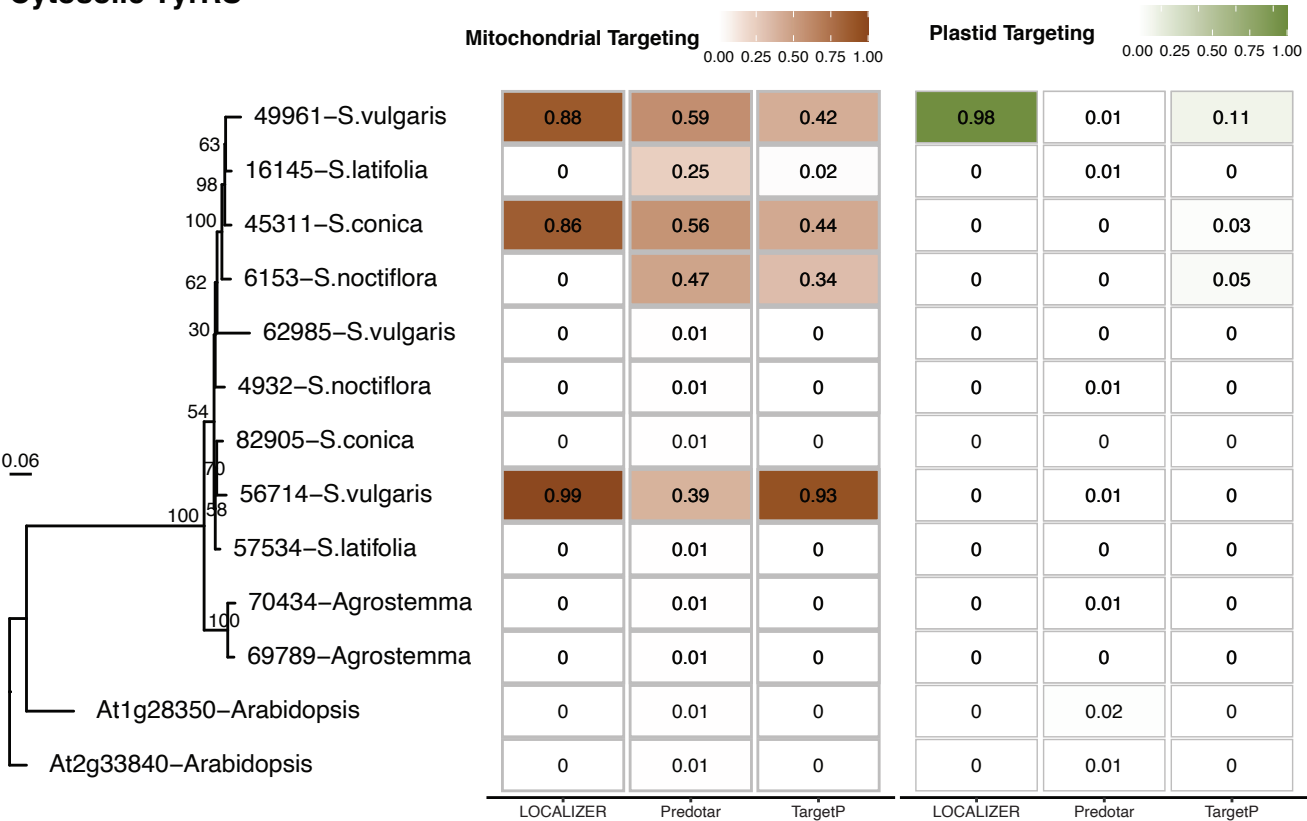

Organellar TyrRS

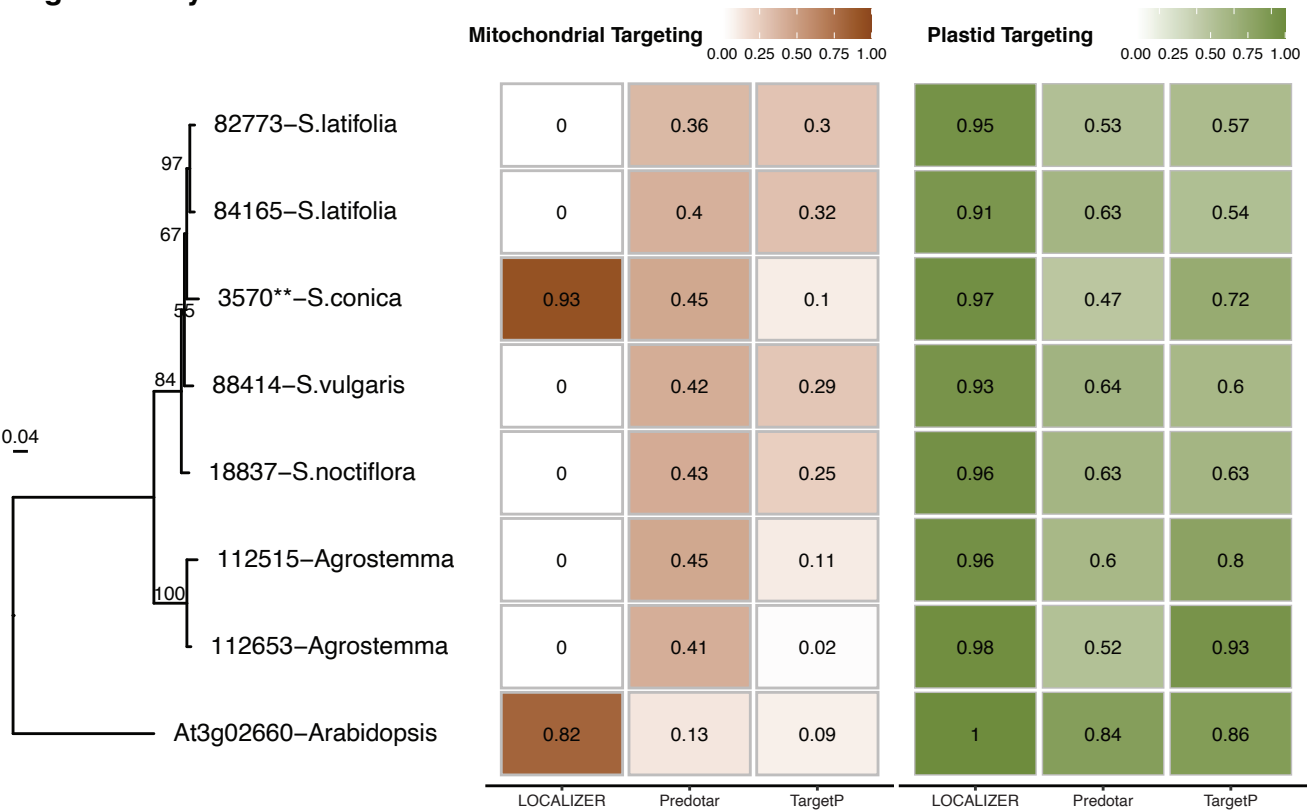

Supplement: msad163_Supplementary_Data [file msad163_supplementary_data.zip › Supp.fig19_TyrRS.pdf]

Cytosolic/Organellar ValRS

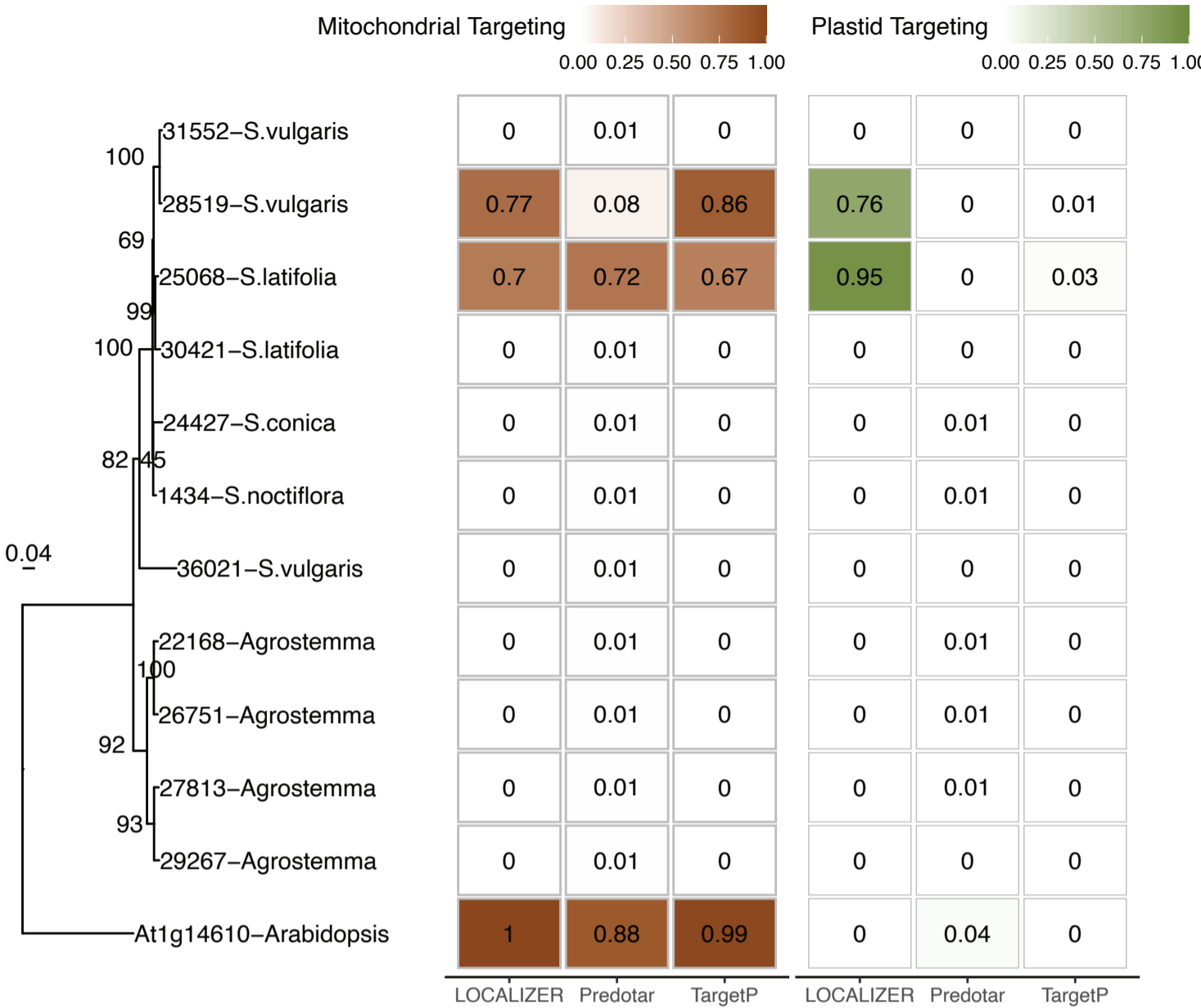

Organellar ValRS

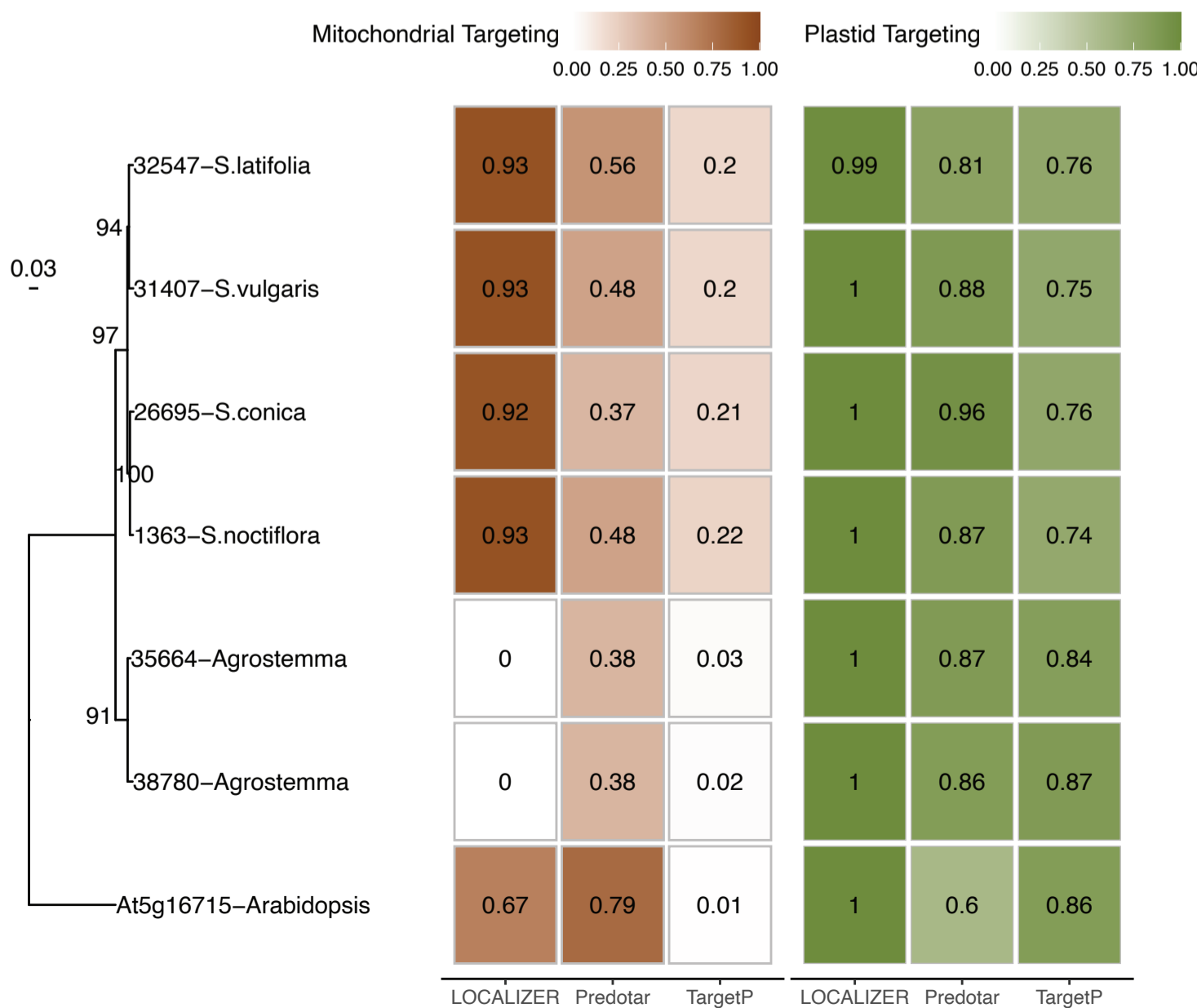

Supplement: msad163_Supplementary_Data [file msad163_supplementary_data.zip › Supp.fig20_ValRS.pdf]
